# An Overview of Secondary Metabolites from Soft Corals of the Genus *Capnella* over the Five Decades: Chemical Structures, Pharmacological Activities, NMR Data, and Chemical Synthesis

**DOI:** 10.3390/md22090402

**Published:** 2024-09-02

**Authors:** Can-Qi Liu, Qi-Bin Yang, Ling Zhang, Lin-Fu Liang

**Affiliations:** College of Chemistry and Chemical Engineering, Central South University of Forestry and Technology, Changsha 410004, China

**Keywords:** marine natural products, marine invertebrates, soft coral, *Capnella*, chemical constituents, capnellane, pharmacological activities, NMR data, chemical synthesis

## Abstract

There has been no specific review on the secondary metabolites from soft corals of the genus *Capnella* till now. In this work, all secondary metabolites from different species of the title genus were described. It covered the first work from 1974 to May 2024, spanning five decades. In the viewpoint of the general structural features, these chemical constituents were classified into four groups: sesquiterpenes, diterpenes, steroids, and lipids. Additionally, the ^1^H and ^13^C NMR data of these metabolites were provided when available in the literature. Among them, sesquiterpenes were the most abundant chemical compositions from soft corals of the genus *Capnella*. A variety of pharmacological activities of these compounds were evaluated, such as cytotoxic, antibacterial, antifungal, and anti-inflammatory activities. In addition, the chemical synthesis works of several representative sesquiterpenes were provided. This review aims to provide an up-to-date knowledge of the chemical structures, pharmacological activities, and chemical synthesis of the chemical constituents from soft corals of the genus *Capnella*.

## 1. Introduction

Soft corals of the subclass Octocorallia (class Anthozoa) are abundant in the ocean worldwide, where they are an important group of contributors to the chemically diverse and biologically active marine natural products [1,2,3,4]. For instance, soft corals of a vast array of genera including *Sarcophyton* [5,6,7], *Sinularia* [8,9,10,11], *Lobophytum* [12,13], *Cladiella* [14], *Litophyton* [15], *Alcyonium* [16], *Dendronephthya* [17], *Xenia* [18], *Verrucella* [19], *Cespitularia* [20], and *Lemnalia* [21] have been extensively studied and furnished with a wealth of secondary metabolites with various structural features including sesquiterpenes, diterpenes, dimeric terpenes, meroterpenes, steroids, ceramides, *etc*. Moreover, these metabolites exhibited a variety of pharmaceutical properties, such as antibacterial [22], promoting angiogenesis [23], anti-inflammatory [24], osteoclastogenesis inhibitory [25], cytotoxic [26,27,28], TNF-*α* inhibitory [29], anti-COVID [30], and antituberculosis [31] effects. Due to their unique complex structures, these compounds gained great attention from synthetic scientists for their total synthesis [32,33].

In our continuous work on the discovery of structurally intriguing molecules from soft corals of different genera [22,29,34,35,36,37,38], we compiled systematic reviews of soft corals [14,15,39] and sponges [40,41] recently. During the process of literature collection of the genus *Litophyton*, we encountered the title genus *Capnella* because both of them were reported to belong to the family Nephtheidae of the subclass Octocorallia [42]. It might be worth pointing out that this genus was recently revisited and revised as a genus of the new family Capnellidae by phylogenomics [43].

The soft corals of the genus *Capnella* strictly inhabit the Indo-Pacific region. These marine organisms offer an enormous source of potentially novel secondary metabolites, such as sesquiterpenes [44], diterpenes [45], steroids [46], and lipids [47]. These chemical constituents showed a library of pharmacological efficacies including cytotoxic [48], antibacterial [49], antifungal [50], and anti-inflammatory [51] activities. Notably, the well-known capnellane sesquiterpenes are the characteristic chemical constituents of this genus, the skeleton name of which was named after the genus name [52]. Featured by the fascinating tricyclic backbone along with the highly complex chirality, the capnellane sesquiterpenes were attractive targets for synthetic scientists for a long time [53,54,55,56], who devoted themselves to the development of different methods to efficiently synthesize them. However, there has been no specific review on the secondary metabolites from soft corals of the genus *Capnella* till now. In this work, all the secondary metabolites reported from different species of this genus from 1974 to May 2024 were summarized, including their pharmacological activities and ^1^H and ^13^C NMR data whenever available. Moreover, the chemical synthesis works of several representative sesquiterpenes were presented.

## 2. An Overview of Secondary Metabolites from Soft Corals of the Genus *Capnella*

**Figure 1:**
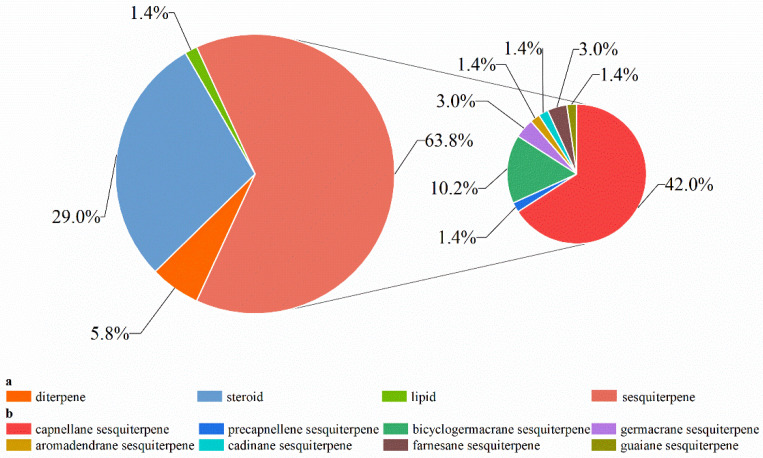
The scales of different types of secondary metabolites from soft corals of the genus *Capnella*: (**a**) general groups as sesquiterpenes, diterpenes, and steroids; (**b**) detail classifications of sesquiterpenes.

This review covers the secondary metabolites from soft corals of the genus *Capnella* from 1974 to May 2024, based on an extensive literature search using SciFinder, Web of Science, and PubMed. As revealed by the literature survey, 69 chemical components were isolated and identified from soft corals of this genus over the five decades (Table 1). These metabolites possessed diverse structural features, which could be generally grouped as sesquiterpenes, diterpenes, steroids, and lipids (Figure 1a). Obviously, sesquiterpenes were the dominant type of chemical composition from soft corals of the genus *Capnella*. In-depth insights into the chemical diversities of sesquiterpenes could be further divided into eight subclasses including capnellane, precapnellane, bicyclogermacrane, germacrane, aromadendrane, cadinene, farnesane, and guaiane (Figure 1b). As shown in Figure 1b, capnellane sesquiterpenes were the most abundant metabolites in this genus.

**Figure 2:**
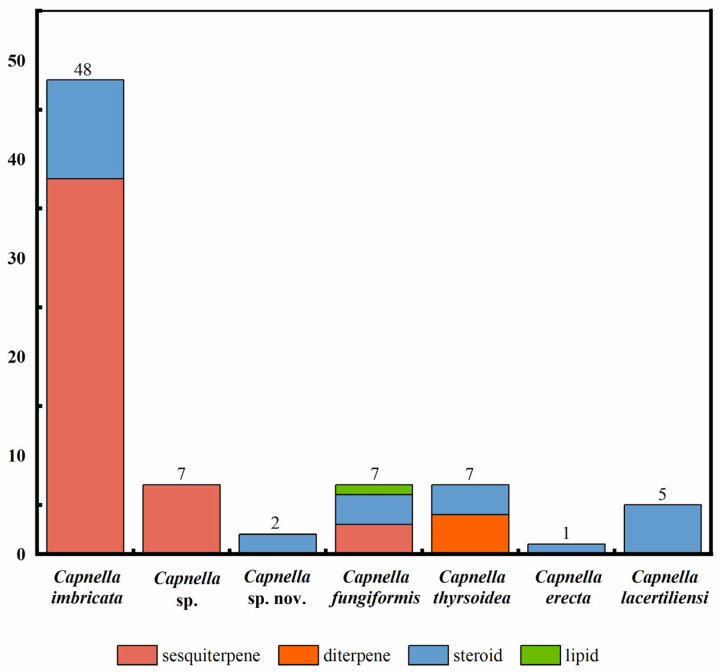
The distributions of secondary metabolites in different species of the genus *Capnella*.

Insights into the distributions of these secondary metabolites in different species of the genus *Capnella*, including *Capnella imbricata*, *Capnella lacertiliensi*, *Capnella thyrsoidea*, *Capnella erecta*, *Capnella fungiformis*, *Capnella* sp. nov., and *Capnella* sp. were reported as their source species (Figure 2). Among these aforementioned species, *C. imbricata* was the most frequently encountered species, and afforded the largest number of secondary metabolites with a total amount of 48. (Notably, some compounds were distributed in different species and they were counted separately in each species). Interestingly, diterpenes were solely found in the species *C. thyrsoidea*, and three species—*C. lacertiliensi*, *C. erecta,* and *Capnella* sp. nov.—only afforded steroids. Perhaps, these secondary metabolites could serve as chemotaxonomic markers for these species.

## 3. Sesquiterpenes

**Figure 3:**
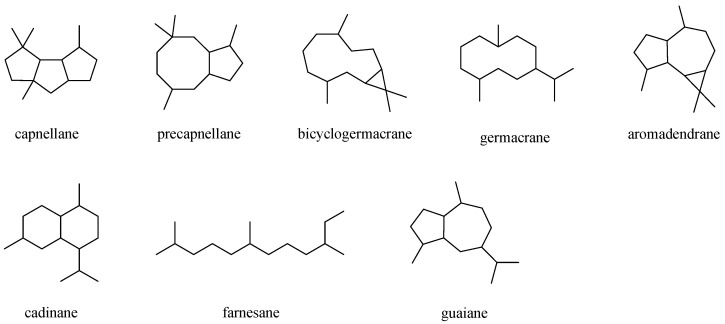
Basic skeletons of sesquiterpenes reported from soft corals of the genus *Capnella*.

This was the largest cluster of secondary metabolites (Figure 1a) obtained from soft corals of the genus *Capnella* with an amount of 45 compounds in this work (Table 1). These compounds showed a diversity of carbon frameworks, which comprised eight categories: capnellane, precapnellane, bicyclogermacrane, germacrane, aromadendrane, cadinene, farnesane, and guaiane (Figure 3). These diverse skeletons make sesquiterpenes the most attractive type of secondary metabolites from this genus. Interestingly, the majority of sesquiterpenes were obtained from the species *C. imbricata*. The rest of the substances were present in the species *C. fungiformis* and the unclearly identified *Capnella* sp. (Table 1).

### 3.1. Capnellane Sesquiterpenes

**Figure 4:**
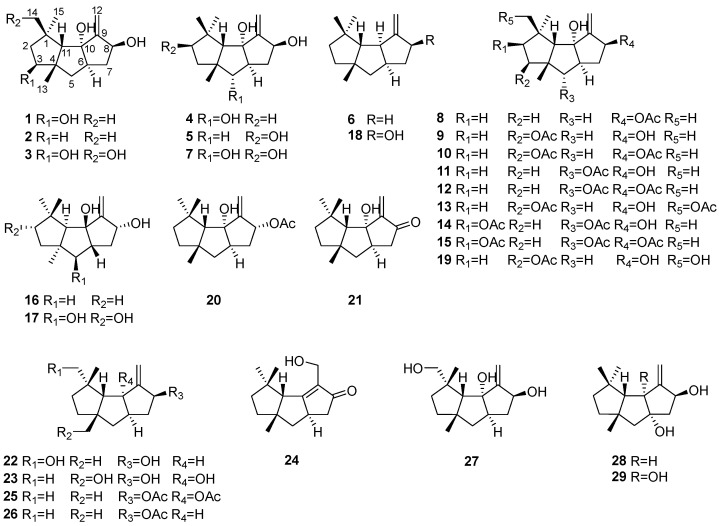
Chemical structures of capnellane sesquiterpenes obtained from soft corals of the genus *Capnella*.

This type of sesquiterpene was the characteristic group of secondary metabolites from soft corals of the genus *Capnella* (Figure 4) with the most abundance (Figure 1b). Worth mentioning was that the skeleton name capnellane was coined by scientists in their work on *C. imbricata* [52]. The basic skeleton of capnellane was the unique tricyclic 5/5/5 carbon framework bearing four methyls including one *gem*-dimethyl and one angular methyl.

In 1974, the first terpene **1** from soft corals of the genus *Capnella* was obtained from the Indonesian soft coral *C. imbricata* from Leti Island. Its structure was elucidated through the extensive analysis of spectral data along with a series of chemical conversions [52]. Importantly, the absolute configuration of **1** was successfully established by single-crystal X-ray diffraction in the subsequent study [57].

Continuing investigation on the Indonesian collection *C. imbricata* afforded two undescribed metabolites **2** [58] and **3** [63], in addition to **1**. The complete structure of **3** was established by a single-crystal X-ray diffraction analysis, and its absolute configuration was identified as the same as that of **1** [63]. Notably, different inhabited environments caused different compositions of secondary metabolites of *C. imbricata*. This research team found that another sample of this species from Lakor Island had two triols **4** and **5**, in addition to the diol **2** but without the tetrol **3** [58]. Meanwhile, hydrocarbon **6** was obtained from the nonpolar fraction of *C. imbricata*. This compound was assumed the biogenetic precursor of this class of sesquiterpene alcohols, which was confirmed by partial synthesis in the work [64].

A new terpene **7** was identified in the Papua New Guinean specimen *C. imbricata*. Its structure and relative configuration were determined by a single-crystal X-ray diffraction analysis. Based on the closely resembling NMR data, the relative stereochemistry at C-2 for previously described **5** was proposed as 2*β* [65]. This speculation was unambiguously confirmed by the diagnostic NOESY correlations and the Gaussian calculation in the recent Chung and co-workers’ research [44]. The presence of eight novel acetylated capnellenes **8**–**15** was described in the fresh colonies of *C. imbricata*, as they were the major sesquiterpenes in the living animal. According to the results of chemical transformations, the previously reported capnellene polyols, isolated from sun-dried colonies, were shown to be artifacts [66].

Chemical investigation of the South China Sea collection *C. imbricata* led to the discovery of two sesquiterpenes **16** [67] and **17** [68]. Their structures were established by extensive analysis of spectral data and confirmed by single-crystal X-ray diffraction experiments. It was found that the signs of the specific rotation values of **16** ([α]_D_ −39.6) [67] and **17** ([α]_D_ −17) [68] were respectively opposite to those of **2** ([α]_D_ +41) [58] and **7** ([α]_D_ +17) [65]. Consequently, these four terpenes could be regarded as two pairs of enantiomers. Preliminary bioassays showed that compound **17** had suppressive action on contracture of the removed ileums of the guinea pig and it exhibited antitumor effects on EAC at 25 μg/mL with an inhibition rate of 43% [68].

Two previously unrecorded compounds with the capnellane skeleton **18** and **19**, along with one known metabolite **2**, had been isolated from the Indonesian sample *C. imbricata* collected at Mayu Island. The cytotoxicities of all the isolates were tested against six cell lines including HL-60, K562, G402, MCF-7, HT115, and A2780. The results showed that compound **2** was cytotoxic toward all tested cell lines with IC_50_ values ranging from 0.7 μM to 93 μM, with the best activity being displayed against K562 leukemia. Sesquiterpene **18** was effective against the cell lines K562 and A2780 (IC_50_ 4.6 μM and 6.6 μM, respectively). Component **19** was cytotoxic with selectivity for cell lines G402 and A2780 [48].

Interestingly, chemical constituents **2** and **18** were also found in the Formosan soft coral *C. imbricata*, along with seven sesquiterpenes **20**–**26**. In this work, their anti-inflammatory activities were evaluated. Compounds **2**, **20,** and **21** significantly reduced the iNOS protein expression (1.2 ± 0.1%, 54.4 ± 12.0%, and 34.8 ± 10.2%, respectively). Moreover, substances **2** and **20** significantly inhibited the expression of the COX-2 protein (24.8 ± 7.5% and 62.9 ± 13.7%, respectively) [51]. Further bioassays were conducted, revealing marine-derived capnellenes **2** and **18** had anti-neuroinflammatory and anti-nociceptive properties in IFN-γ-stimulated microglial cells and in neuropathic rats, respectively [59]. Another Formosan soft coral *Capnella* sp. afforded the secondary metabolite **2**, too. This compound together with its 10 ester derivatives were tested for cytotoxic activities against four human tumor cell lines KB, WiDr, Hela, and Daoy. Almost all esters showed superior activity against these cells compared to the parent compound **2** [61]. In recent pharmacological research, substance **2** impaired vascular development in zebrafish [60].

Study on another Formosan collection *C. imbricata* afforded a new secondary metabolite **27** along with two known metabolites **2** and **4**. In this study, a single-crystal X-ray diffraction analysis of **4** was conducted for the first time. Compound **27** exhibited significant inhibitory effects on elastase release and superoxide generation with inhibition rate of 5.67% and 9.28%, respectively [62]. Further investigation resulted in the discovery of two new sesquiterpenes **28** and **29**, as well as **4**, **5**, **18**, **20**, and **27**. In the anti-inflammatory activity bioassay, components **20** and **29** showed remarkable decreases in iNOS levels (47.61% and 27.73%, respectively). Furthermore, terpenes **4**, **20**, and **28** exhibited significant inhibitions against COX-2 protein expressions (ranging from 7.64% to 12.57%). Notably, the chemical relationships between these sesquiterpenes and their anti-inflammatory effects was analyzed by a tool called ChemGPS-NP [44].

To the best of our knowledge, there was only one report of natural capnellane sesquiterpenes from other organisms, which did not belong to the genus *Capnella*. This was the investigation on the soft coral *Dendronephthya rubeola*, resulting in the isolation and identification of two aforementioned metabolites **2** and **8**, along with four undescribed capnellane sesquiterpenes [77]. It might be worth pointing out that the illihenlactoneosides B and C from the plant *Illicium henryi* [78] were not capnellane-type sesquiterpenes, because their tricyclic carbon framework did not bear the key *gem*-dimethyl substructure. Based on these findings, all the capnellane sesquiterpenes except **2** and **8** were characteristic chemical constituents of *Capnella* soft corals.

### 3.2. Precapnellane Sesquiterpene

**Figure 5:**
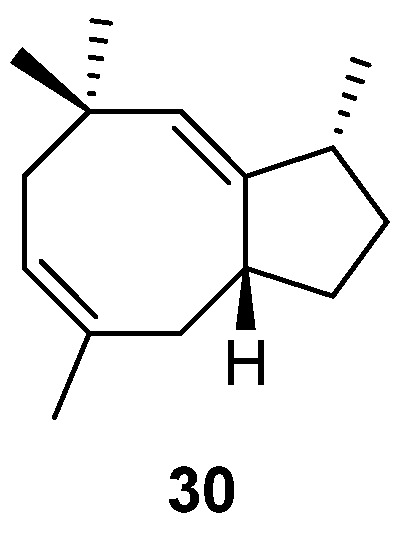
Chemical structure of the precapnellane sesquiterpene obtained from soft corals of the genus *Capnella*.

The sole member of this group was precapnelladiene (**30**) (Figure 5) from the soft coral *C. imbricata*. The basic precapnellane carbon framework had a fused 5- and 8-membered ring system forming an uncommon bicyclo[6.3.0]undecane carbon skeleton, carrying four methyls including one *gem*-dimethyl. From the biogenetic viewpoint, this type of compound might be a precursor of the co-occurring tricyclic capnellane sesquiterpenes [69].

As far as we know, there was only one additional member of precapnellane sesquiterpene called 3*α*,4*α*-epoxyprecapnell-9(12)-ene, which was afforded by the soft coral *D. rubeola* [77]. However, there was no further report of **30** from other biological materials. As a result, carbonhydron **30** could be regarded as the representative chemical substance of *Capnella* soft corals.

### 3.3. Bicyclogermacrane Sesquiterpenes

The bicyclogermacrane contains a bicyclo[8.1.0]undecane carbon skeleton, featured by a cyclopropane fused to a cyclodecane and the *gem*-dimethyl attached to the cyclopropane.

**Figure 6:**
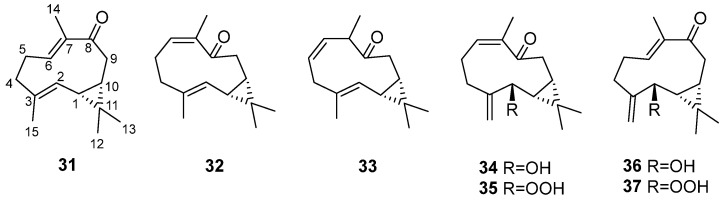
Chemical structures of bicyclogermacrane sesquiterpenes obtained from soft corals of the genus *Capnella*.

The first reported bicyclogermacrane sesquiterpenes from soft corals of the genus *Capnella* were capgermacrenes A (**31**) and B (**32**) (Figure 6). Interestingly, these two terpenes were a pair of isomers that differed in the configuration of double bond Δ^6^. However, only **31** exhibited anti-inflammatory activity, which was possibly influenced by the stereo difference [70]. Continuous study on the same species afforded another new metabolite **33**, whose double bond was shifted to C-5/C-6 compared to **31** and **32**. However, it did not show any obvious antibacterial activity against *Escherichia coli* and *Staphylococcus aureus* [72].

Investigation on another Bornean soft coral *C. imbricata* yielded four new members of bicyclogermacrane sesquiterpenes **34**–**37**. These four chemical constituents were the respective hydroxyl or hydroperoxyl derivatives of **31** and **32**. Terpenes **34** and **35** displayed bacteriostatic activity against *S. aureus* and MRSA, while components **36** and **37** exhibited bactericidal activity against these two bacteria [49]. Indeed, the co-existence of capgermacrenes A–G (**31**–**37**) was found in the soft coral *C. imbricata*. All of these metabolites except **33** exhibited different levels of cytotoxicity against S1T cells with IC_50_ values ranging from 0.79 μg/mL to 7.79 μg/mL [71].

Literature surveys revealed that these seven compounds have not been found in the other organisms except the aforementioned soft corals. Consequently, they served as the featured secondary metabolites of *Capnella* soft corals.

### 3.4. Germacrane Sesquiterpenes

The germacrane contains a cyclodecane bearing one isopropyl and two methyls. Formally, this could be regarded as the precursor of bicyclogermacrane.

**Figure 7:**
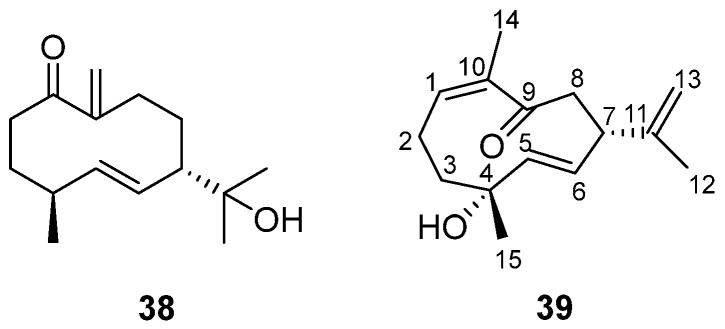
Chemical structures of germacrane sesquiterpenes obtained from soft corals of the genus *Capnella*.

Litseagermacrane (**38**) (Figure 7) was a germacrane sesquiterpene afforded by the Bornean soft coral *Capnella* sp. However, no obvious anti-inflammatory activity was observed in the bioassay [70]. Another study on the Bornean soft coral *C. imbricata* resulted in the discovery of a new germacrane sesquiterpene called capgermacrene H (**39**). This compound was inactive against S1T cells (IC_50_ > 30.0 μg/mL) [71].

Interestingly, compound **38** was also obtained from three plants *Litsea verticillata* [79], *Dysoxylum mollissimum* [80], and *Syzygium cerasiforme* [81]. However, there was no report of metabolite **39** from other marine organisms besides the above-mentioned soft corals. Therefore, **39** could be regarded as the characteristic chemical composition of *Capnella* soft corals.

### 3.5. Aromadendrane Sesquiterpene

The basic carbon skeleton of aromadendrane is the non-linear fused tricyclic 5/7/3 carbon framework. There are four methyls attached to the carbon skeleton, including one *gem*-dimethyl at the cyclopropane.

**Figure 8:**
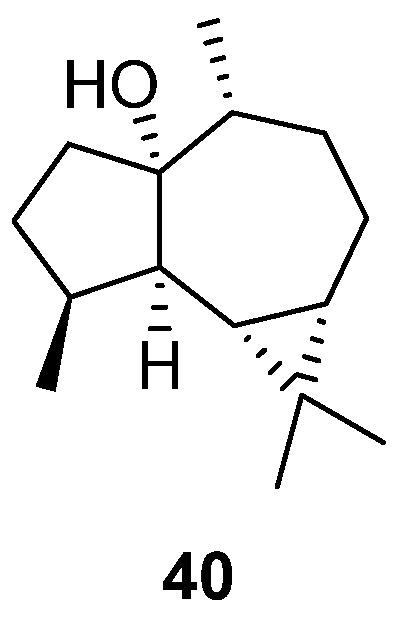
Chemical structure of the aromadendrane sesquiterpene obtained from soft corals of the genus *Capnella*.

Chemical investigation of Bornean soft coral *Capnella* sp. led to the isolation and identification of the aromadendrane sesquiterpene **40** (Figure 8), which was inactive in the anti-inflammatory bioassay [70].

It might be worth pointing out that this sesquiterpene was widely distributed in the soft corals including *Sarcophyton glaucum* [82], *Sarcophyton trocheliophorum* [83], and *Xenia umbellate* [84], and plants such as *Artemisia vulgaris* [85], *Turczaninowia fastigiate* [86], *Hyptis mociniana* [87], *Calycolpus goetheanus* [88], and *Calypogeia integristipula* [89]. As a result, this compound had no chemotaxonomic significance.

### 3.6. Cadinane Sesquiterpene

The decalin is the basic carbon skeleton of cadinene, and one isopropyl and two methyls are attached to it. Structurally, one methyl and one isopropyl are in the *para* position.

**Figure 9:**
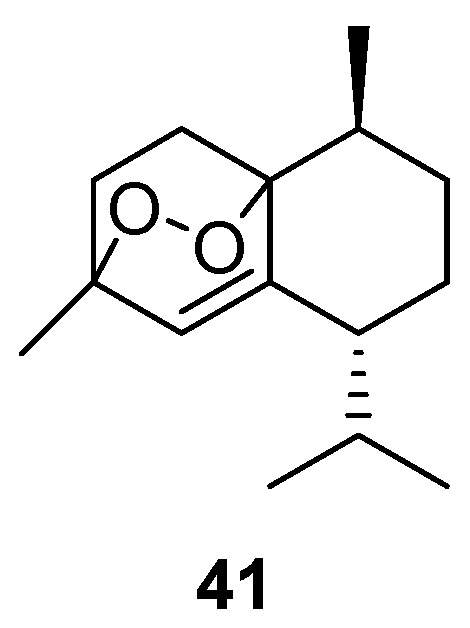
Chemical structure of the cadinane sesquiterpene obtained from soft corals of the genus *Capnella*.

The Bornean soft coral *Capnella* sp. afforded the cadinane sesquiterpene **41** (Figure 9). The antibacterial bioassay revealed the negligible inhibition against *E. coli* and *S. aureus* (MIC > 500 μg/mL) [72].

This substance was previously found in the soft coral *Sarcophyton ehrenbergi* [90] and the plant *Scapania undulata* [91]. Interestingly, the enantiomer of **41** was encountered in the investigation of soft coral *Sinularia* sp. [92]. These reports revealed that **41** could not be the typical component of *Capnella* soft corals.

### 3.7. Farnesane Sesquiterpenes

The farnesane is a linear carbon chain, which is formed by the head-to-tail connection of three isoprene units.

**Figure 10:**
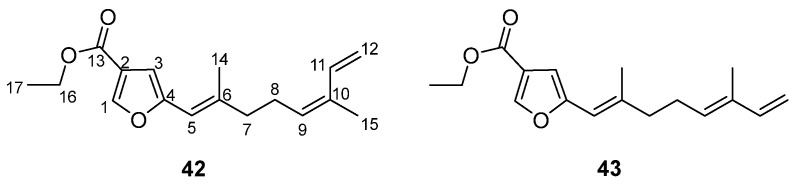
Chemical structures of farnesane sesquiterpenes obtained from soft corals of the genus *Capnella*.

A study on the Madagascan soft coral *C. fungiformis* gave an inseparable mixture of *Z*/*E*-isomers **42** and **43** (Figure 10), which was determined by GC-MS and NMR spectral data [47].

It might be worth pointing out that the carboxylic acids and methyl esters of these two metabolites were reported as the isolates of soft corals *Sinularia gonatodes* [93], *Sinularia capillosa* [94], *Sinularia kavarattiensis* [95,96] and *Sinularia tumulosa* [22]. Although they were new natural products, it was difficult to evaluate whether they were characteristic secondary metabolites of *Capnella* soft corals.

### 3.8. Guaiane Sesquiterpene

The guaiane contains a bicyclo[5.3.0]decane carbon skeleton carrying one isopropyl and two methyls. Formally, this could be regarded as the precursor of aromadendrane.

**Figure 11:**
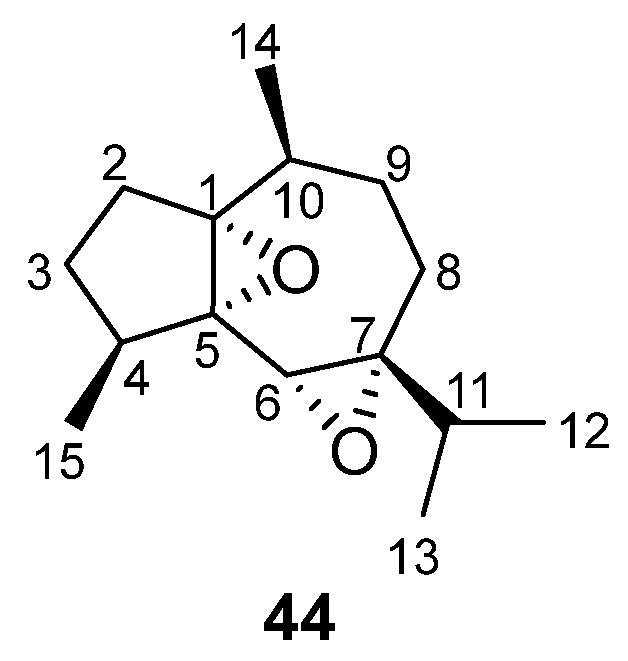
Chemical structure of guaiane sesquiterpene obtained from soft corals of the genus *Capnella*.

A novel guaiane sesquiterpene oxyfungiformin (**44**) (Figure 11) was isolated from the soft coral *C. fungiformis*. Its structure was elucidated by detailed analysis of spectral data and quantum chemical calculations [47]. Recently, its stereochemistry was confirmed by the synthesis work, along with the determination of its absolute configuration by X-ray diffraction [97].

To the best of our knowledge, only one structurally related compound was reported from the soft coral *S. kavarattiensis* [96], which was a diastereoisomer of **44** at the opposite geometry of 1,5-epoxy. Consequently, **44** was a specific chemical constituent of *Capnella* soft corals.

## 4. Diterpenes

**Figure 12:**
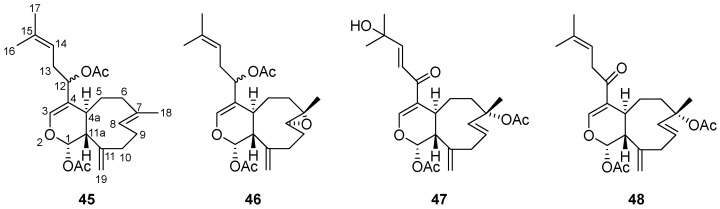
Chemical structures of xenicane diterpenes obtained from soft corals of the genus *Capnella*.

Unlike soft corals of some frequently encountered genera and species [7,11,14,15,16,39], diterpenes were extremely rare in the genus *Capnella*. To the best of our knowledge, only four diterpenes were found. They were xenicane diterpenes **45**–**48** (Figure 12), from the South African sample *C. thyrsoidea* [45]. Good inhibitory activity against superoxide production in rabbit neutrophils (>80%) at a concentration of 12.5 μg/mL for terpenes **45**, **47,** and **48,** and good to moderate inhibition of superoxide production in human neutrophils (68% and 21%, respectively) at a concentration as low as 1.25 μg/mL for **45** and **47** were observed.

The xenicane featured a cyclononane ring, and usually, a tetrahydropyran ring bearing a long carbon chain fuses to the aforementioned macro ring. It might be worth pointing out that the 6*β*-epimer of **45**, which was named 9-deacetoxy-14,15-deepoxyxeniculin, was afforded by the soft corals *Xenia obscuronata* [98] and *Eleutherobia aurea* [99], as well as the plant *Boerhavia diffusa* [100]. However, all of these four compounds have not been obtained from other natural sources. Therefore, they were characteristic chemical components of *Capnella* soft corals.

## 5. Steroids

**Figure 13:**
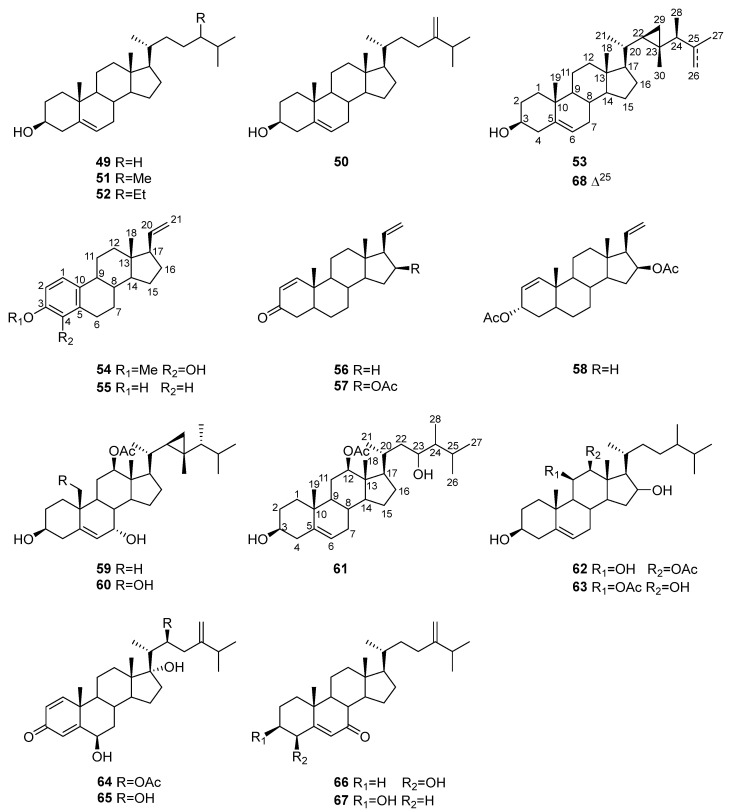
Chemical structures of steroids obtained from soft corals of the genus *Capnella*.

An account of 20 compounds were reported from soft corals of the genus *Capnella* (Figure 13). These steroids could be divided into three groups: ergostane, pregnane, and gorgostane. The major difference between them is the features of the side chain. For pregnane, its side chain is usually vinyl. For gorgostane, its side chain possesses cyclopropane. The left compounds belong to the ergostane steroids.

Analysis of complex sterol mixtures of *C. imbricata* by the mass-analyzed ion kinetic energy spectrometry led to the identification of five sterols **49**–**53** [73]. Recently, the presence of steroids **50**, **51**, and **53** in the Madagascan soft coral *C. fungiformis* was reported [47].

Pregnane sterols **54**–**56** from Australian soft corals *Capnella* sp. nov. and *C. erecta* were illustrated. Notably, the structure of **54** was confirmed by a single-crystal X-ray diffraction [74]. It might be worth pointing out that compound **56** was also obtained from the soft coral *Sinularia* sp., whose structure was also corroborated by the X-ray diffraction analysis [101].

The South African soft coral *C. thyrsoidea* also afforded pregnane steroid **56** together with two new compounds **57** and **58**. It was found that components **56** and **57** stimulated superoxide production in rabbit neutrophils [45]. Additionally, a structure-based virtual docking study suggested **56** as a potential hit with inhibitory activity against plasmodial proteases and selectivity on human cathepsins [75].

Five highly oxygenated sterols (gorgosterols **59** and **60** and ergosterols **61**–**63**) were isolated from the Australian soft coral *C. lacertiliensis*. All sterols displayed the antifungal potential against *Microbotryum violacea*. Moreover, steroids **59** and **63** showed inhibition against *Eurotium repens*, whereas **61** and **63** exhibited weak tyrosine kinase p56*^lck^* inhibition [50].

Recently, the Formosan soft coral *C. imbricata* afforded two new sterols **64** and **65** with a cross-conjugated dienone structural unit in ring A. These two metabolites showed reduction in iNOS levels and promotion in COX-2 release [46]. Further study on this species yielded three steroids **66**–**68**. In this study, the absolute configuration of sterol **68** was determined for the first time by single-crystal X-ray diffraction analysis. The effects of a moderate reduction in iNOS levels for secondary metabolites **66**–**68** were observed [76].

It is well known that steroids are one enormous cluster of secondary metabolites from soft corals [2,4]. Among the aforementioned steroids, some of the known compounds were frequently encountered in the investigations of soft corals of various species. For instance, compound **50** was found as a metabolite of soft corals such as *Sinularia depressa* [102], *Sinularia nanolobata* [103], *Sinularia humilis* [104], *Litophyton arboreum* [105], *Sinularia terspilli* [106], *Nephthea erecta* [107], *Sinularia flexibilis* [108], *Dendronephthya* sp. [109], *Sinularia gibberosa* [108], *Litophyton viridis* [110], *Sarcophyton glaucum* [111], *etc*. Accordingly, their wide distribution limited their chemotaxonomic significance. However, those new compounds have hardly been reported from other natural sources. Therefore, most of them could be considered as the specific chemical constituents of *Capnella* soft corals.

## 6. Lipid

**Figure 14:**
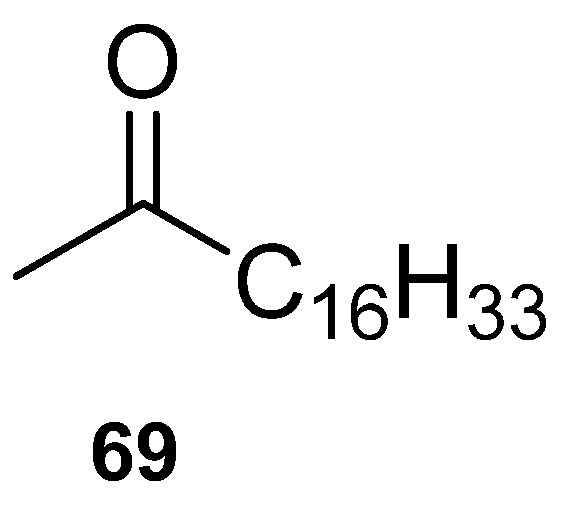
Chemical structure of the lipid obtained from soft corals of the genus *Capnella*.

The lipid **69** was isolated from the Madagascan soft coral *C. fungiformis* (Figure 14) [47].

This compound was found in various natural sources, including the soft coral *Dendronephthya hemprichi* [112], the terrestrial plants *Euonymus latifolius* [113], *Trichilia gilgiana* [114], and *Pinus massoniana* [115], and the marine seaweeds *Codium tomentosum* and *Plocamium cartilagineum* [116]. Its widespread distribution limited the possibility as a featured component of *Capnella* soft corals.

## 7. The Preliminary Summary of Structure-Activity Relationships of the Terpenes from Soft Corals of the Genus *Capnella*

### 7.1. Sesquiterpenes

#### 7.1.1. Capnellane Sesquiterpenes

The capnellane sesquiterpenes were evaluated mainly for cytotoxic and anti-inflammatory activities as depicted in Table 1.

A comparison of **2** and **18** showed the substitution of 10-hydroxyl in **2** could broaden the cytotoxicity against different cell lines [48]. The observed assessment revealed that the presence of hydroxyl groups in C-5 and C-10 in **4** significantly inhibited COX-2 protein expression at a concentration of 10 µM, as compared with **18** [44]. Interestingly, acetylation of 8-hydroxyl and the remaining 10-hydroxyl in 20 still kept the remarkable inhibition against the expression of COX-2 [44]. It was likely that the oxidation of 8-hydroxyl in **21** and **24** led to the loss of reduction of iNOS and COX-2 expression [51]. Seemingly, the substituted 6-hydroxyl in **28** promoted the COX-2 expression, whereas 2-hydroxyl in **30** reduced the expression of COX-2 [44].

#### 7.1.2. Bicyclogermacrane Sesquiterpenes

The seven bicyclogermacrane sesquiterpenes **31**–**37** were subjected to anti-inflammatory, antibacterial, and cytotoxic activities as shown in Table 1.

A comparison of **31** and **32** revealed that the Z configuration of double bond Δ^6^ in **32** significantly reduced the anti-inflammatory activity [70]. Additionally, the stereo difference of Δ^6^ in **34**–**37** had an impact on the antibacterial properties [49]. The shift of Δ^6^ to Δ^5^ in **33** resulted in the loss of antibacterial activity against *E. coli* and *S. aureus* [72] and cytotoxicity activity against SIT cells [71]. For the bioactive compounds **34**–**37**, the change of hydroxyl and hydroperoxyl groups did not improve cytotoxicity [71].

### 7.2. Diterpenes

The result of anti-inflammatory bioassay of four xenicane diterpenes **45**–**48** indicated that the presence of the epoxide in **46** led to the loss of anti-inflammatory activity [45]. A comparison of **48** and **49** indicated that the substitution of a hydroxyl group at C-15 in **47** slightly improved the anti-inflammatory activity at a low concentration.

## 8. The Characteristic ^1^H and ^13^C NMR Data of the Secondary Metabolites from Soft Corals of the Genus *Capnella*

Among these secondary metabolites from soft corals of the genus *Capnella*, a large quantity of them were discovered from natural sources for the first time. Herein, the characteristic ^1^H and ^13^C NMR data of the new chemical constituents were provided in the following tables. However, the NMR data of known compounds such as **20**, **21**, **39**, **41,** and **42** were not given in the literature. Hopefully, the available information of NMR data will be useful for readers who are experts in the structural determination of natural products.

Based on their structural features, the reported ^1^H and ^13^C NMR data of new metabolites were present as described below.

### 8.1. Sesquiterpenes

#### 8.1.1. Capnellane Sesquiterpenes

The capnellane sesquiterpenes were the most abundant sesquiterpenes from soft corals of the genus *Capnella*. Notably, almost all of them were found as novel compounds. It might be worth pointing out that the NMR data of some new compounds such as **7**, **8**, **10**, **12**, and **15** were not given in their corresponding works. Additionally, the full assignments of the ^1^H and/or ^13^C NMR data of a few chemical substances such as **1**–**3** were not completed in their very early investigations; thus, these data were presented as a paragraph instead of a table. The ^1^H and ^13^C NMR data of the left compounds were provided in Table 3, Table 4, Table 5, Table 6 and Table 7.

Compound **1**: ^1^H NMR (CDCl_3_): *δ*_H_ 5.38 (d, *J* = 1.5 Hz, 2H), 4.83 (m, 1H), 4.10 (dd, *J* = 11.0, 6.5 Hz, 1H), 1.28 (s, 3H), 1.20 (s, 3H), 1.01 (s, 3H) [52]; ^1^H NMR (CDCl_3_): *δ*_H_ 5.38 (d, *J* = 1.5 Hz, 2H), 4.83 (m, 1H), 4.13 (dd, *J* = 6, 5 Hz, 1H), 1.28 (s, 3H), 1.20 (s, 3H), 1.05 (s, 3H) [58]; ^13^C NMR (CDCl_3_): see Table 2. 

Compound **2**: ^1^H NMR (CDCl_3_): *δ*_H_ 5.33 (br s, 1H), 5.31 (br s, 1H), 4.80 (m, 1H), 1.26 (s, 3H), 1.24 (s, 3H), 1.08 (s, 3H) [58]; ^13^C NMR (CDCl_3_): see Table 2.

Compound **3**: ^1^H NMR (C_5_D_5_N/D_2_O 1:1): *δ*_H_ 5.40 (m, 2H), 5.33 (m, 1H), 4.60 (m, 1H), 3.82 (dd, 2H), 1.68 (s, 3H), 1.42 (s, 3H) [63]; ^13^C NMR (CD_3_OD): see Table 2.

Compound **4**: ^1^H NMR (CD_3_OD/CD_3_COCD_3_ 1:1): *δ*_H_ 5.20 (m, 2H), 4.80 (m, 1H), 3.86 (d, *J* = 5.5 Hz, 1H), 1.32 (s, 3H), 1.18 (s, 3H), 1.12 (s, 3H) [58]; ^13^C NMR (CDCl_3_): see Table 2.

Compound **5**: ^1^H NMR (CDCl_3_): *δ*_H_ 5.37 (m, 1H), 4.70 (m, 1H), 3.70 (m, 1H), 1.45 (s, 3H), 1.28 (s, 3H), 1.16 (s, 3H) [58]; the recent full assignments of ^1^H and ^13^C NMR (CDCl_3_): see Table 3.

Compound **6**: ^1^H NMR (CDCl_3_): *δ*_H_ 4.94 (br s, 1H), 4.82 (br s, 1H), 1.16 (s, 3H), 1.08 (s, 3H), 0.98 (s, 3H) [64]; ^13^C NMR (CDCl_3_): *δ***_C_** 158.75, 105.08, 69.12, 53.39, 52.34, 48.00, 46.07, 42.37, 41.73, 40.63, 31.83, 31.56, 30.84, 29.11, 26.07 [64].

Compound **9**: ^1^H NMR (CDCl_3_): *δ*_H_ 5.36 (br s, 2H), 5.1 (dd, *J* = 12.5, 8 Hz, 1H), 4.8 (m, 1H), 1.95 (s, 3H), 1.32 (s, 3H), 1.24 (s, 3H), 0.98 (s, 3H) [66].

Compound **11**: ^1^H NMR (CDCl_3_): *δ*_H_ 5.38 (br s, 2H), 4.8 (m, 1H), 4.6 (d, *J* = 6.5 Hz, 1H), 2.03 (s, 3H), 1.21 (s, 3H), 1.12 (s, 3H), 1.12 (s, 3H) [66].

Compound **13**: ^1^H NMR (CDCl_3_): *δ*_H_ 5.18 (br s, 2H), 5.0 (dd, *J* = 9, 9 Hz, 1H), 4.68 (m, 1H), 3.96 (d, *J* = 10.5 Hz, 1H), 2.03 (s, 3H), 1.94 (s, 3H), 1.28 (s, 3H), 0.88 (s, 3H) [66].

Compound **14**: ^1^H NMR (CDCl_3_): *δ*_H_ 5.43 (br s, 2H), 4.85 (dd, *J* = 6, 6 Hz, 1H), 4.8 (m, 1H), 4.66 (d, *J* = 5 Hz, 1H), 2.06 (s, 3H), 2.03 (s, 3H), 1.25 (s, 3H), 1.22 (s, 3H), 1.12 (s, 3H) [66].

**Table 2:** The ^13^C NMR data of compounds **1**–**4**.

No.	1 [58]	2 [58]	3 [63]	4 [58]
	*δ*_C_ ^1^	*δ*_C_ ^1^	*δ*_C_ ^2^	*δ*_C_ ^1^
1	38.6	43.3	44.10	43.7
2	51.7	42.7	46.66	42.4
3	81.4	41.4	81.01	32.9
4	52.6	49.3	52.42	53.2
5	45.3	45.6	45.95	82.8
6	49.8	48.7	50.10	56.1
7	38.1	36.8	38.44	34.6
8	73.8	72.4	73.84	72.4
9	161.5	160.3	161.90	159.9
10	89.8	88.8	89.11	86.0
11	65.5	64.6	63.54	64.0
12	109.1	107.5	109.09	108.4
13	25.0	31.5	24.80	30.8
14	32.9	30.3	73.84	31.4
15	26.1	23.2	21.96	24.1

^1^ Recorded in CDCl_3_. ^2^ Recorded in CD_3_OD.

**Table 3:** The ^1^H and ^13^C NMR data of compounds **5**, **16,** and **17**.

No.	5 [44]		16 [67]		17 [68]	
	*δ*_H_ ^1^	*δ*_C_ ^1^	*δ*_H_ ^2^	*δ*_C_ ^2^	*δ*_H_ ^2^	*δ*_C_ ^2^
1		46.9		43.94		47.15
2	4.03 dd (5.4, 5.4)	82.2	1.55 m	43.17	3.57 dd (5.0, 4.0)	83.37
			1.46m			
3	2.09 dd (13.8, 5.4)	50.0	1.75 m	41.98	2.11 m	39.74
	1.55 dd (13.8, 5.4)		1.57 m		2.15 m	
4		47.6		49.94		50.91
5	1.48 m	46.8	1.95 dd (9.0, 4.5)	46.22	3.20 d (1.0)	81.40
	2.04 dd (13.8, 8.4)		1.36 dd (7.4, 6.0)			
6	2.34 m	51.1	2.51 m	49.61	2.35 m	57.04
7	2.32 m	38.1	1.48 ddd (15.2, 9.0, 4.8)	37.88	1.40 m	35.76
	1.50 m		2.35 ddd (10.0, 6.0, 2.6)		2.28 m	
8	4.74 m	73.7	4.80 m	73.51	4.62 m	72.00
9		162.2		162.25		162.00
10		90.5		90.13		85.12
11	2.17 s	64.7	1.46 m	65.69	2.02 s	62.05
12	5.34 d (1.8)	110.3	5.32 d (2.0)	109.51	5.15 m	107.21
	5.39 d (1.8)		5.30 d (2.0)			
13	1.27 s	34.4	1.35 s	32.71	1.03 s	32.80
14	1.11 s	24.2	1.09 s	31.40	1.11 s	25.33
15	1.27 s	23.0	1.26 s	24.08	1.10 s	23.70

^1^ Recorded in CDCl_3_. ^2^ The deuterated solvent was not given.

**Table 4:** The ^1^H and ^13^C NMR data of compounds **18** and **19**.

No.	18 [48]		19 [48]	
	*δ*_H_ ^1^	*δ*_C_ ^1^	*δ*_H_ ^1^	*δ*_C_ ^1^
1		42.5		43.4
2	1.51 m	41.5	1.68 dd (11.2, 5.6)	43.8
			1.55 t (11.2)	
3	2.09 m	40.5	5.08 dd (10.6, 5.6)	82.1
	1.45 m			
4		53.3		51.4
5	1.79 dd (13.8, 8.4)	48.9	2.25 m	45.6
	1.51 dd (13.8, 4.8)		1.20 m	
6	2.22 ddt (7.6, 3.5, 4.2)	42.1	2.52 m	49.2
7	2.13 dd (8.4, 4.2)	40.3	2.35 m	38.3
	1.39 m		1.42 dt (14.4, 4.0)	
8	4.74 tt (8.0, 4.2)	75.6	4.75 m	73.5
9		160.6		161.8
10	2.36 ddd (4.6, 2.8, 1.6)	49.5		87.9
11	1.75 d (3.3)	68.0	2.30 s	64.9
12	5.05 t (2.5)	105.4	5.30 t (2.5)	109.7
	4.96 t (2.4)			
13	1.24 s	32.1	0.82 s	25.3
14	1.02 s	30.8	3.55 d (9.4)	74.1
			3.42 d (9.4)	
15	0.82 s	26.1	1.31 s	20.8
*CO*CH_3_				171.1
CO*CH_3_*			1.99 s	21.0

^1^ Recorded in CDCl_3._

**Table 5:** The ^1^H and ^13^C NMR data of compounds **22**–**24**.

No.	22 [51]		23 [51]		24 [51]	
	*δ*_H_ ^1^	*δ*_C_ ^1^	*δ*_H_ ^1^	*δ*_C_ ^1^	*δ*_H_ ^1^	*δ*_C_ ^1^
1		47.2		44.5		44.7
2	1.41 m	36.2	1.46 m	43.9	1.56 m	42.3
	1.58 m				1.66 m	
3	1.42 m	39.8	1.69 m	37.5	1.78 m	40.4
4		53.6		55.5		55.2
5	1.49 m	48.8	1.60 m	41.5	1.03 dd (12.3, 4.5)	46.2
	1.80 dd (12.8, 8.1)		1.88 dd (14.2, 10.1)		2.09 dd (12.3, 8.0)	
6	2.43 m	41.6	2.67 m	49.5	3.02 m	44.1
7	1.39 m	39.7	1.59 m	38.2	2.05 dd (18.4, 2.0)	42.2
	2.25 m		2.28 m		2.64 dd (18.4, 6.5)	
8	4.51 t (5.2)	75.5	4.78 m	74.2		211.3
9		160.2		164.3		135.5
10	2.86 m	49.1		90.6		186.7
11	1.89 d (3.2)	65.2	2.02 s	62.5	2.47 s	61.9
12	5.00 s	105.9	5.41 s	111.8	4.32 dd (13.4, 1.7)	56.8
	5.14 s		5.43 s		4.37 dd (13.4, 1.7)	
13	1.23 s	31.6	3.35 m	71.4	1.24 s	30.1
14	1.15 s	26.7	1.21 s	30.7	1.21 s	31.1
15	3.48 d (10.8)	69.9	1.29 s	23.8	0.87 s	26.0
	3.58 d (10.8)					

^1^ Recorded in CDCl_3._

**Table 6:** The ^1^H and ^13^C NMR data of compounds **25** and **26**.

No.	25 [51]		26 [51]	
	*δ*_H_ ^1^	*δ*_C_ ^1^	*δ*_H_ ^1^	*δ*_C_ ^1^
1		44.3		43.9
2	1.45 m	43.6	1.44 m	41.7
3	1.54 m	41.7	1.53 m	40.9
	1.64 m			
4		50.2		53.6
5	1.26 dd (9.5, 13.8)	45.3	1.45 m	48.4
	1.83 dd (9.5, 13.8)		1.77 m	
6	2.77 m	48.1	2.48 m	42.6
7	1.47 m	35.6	1.54 m	36.3
	2.51 m		2.25 m	
8	5.72 m	75.4	5.53 t (3.4)	76.2
9		151.5		156.3
10		95.7	2.71 m	49.5
11	2.34 s	65.3	1.78 m	67.7
12	5.45 d (2.2)	116.4	4.99 s	108.6
	5.51 d (2.2)		5.08 s	
13	1.09 s	31.9	1.19 s	31.6
14	1.11 s	31.4	1.06 s	30.2
15	1.12 s	24.4	0.98 s	25.5
8-*CO*CH_3_		170.9		171.9
8-CO*CH_3_*	2.07 s	21.3	2.09 s	21.4
10-*CO*CH_3_		169.6		
10-CO*CH_3_*	1.95 s	22.0		

^1^ Recorded in CDCl_3_.

**Table 7:** The ^1^H and ^13^C NMR data of compounds **27**–**29**.

No.	27 [62]		28 [44]		29 [44]	
	*δ*_H_ ^1^	*δ*_C_ ^1^	*δ*_H_ ^1^	*δ*_C_ ^1^	*δ*_H_ ^1^	*δ*_C_ ^1^
1		49.9		42.1		44.2
2	1.77 dd (12.6, 6.6)	38.3	1.47 m	40.6	1.34 m	41.0
	1.41 dt (12.6, 7.2)				2.05 m	
3	1.65 m	41.9	1.64 m	41.3	1.71 dd (8.0, 2.5)	42.4
	1.62 m				1.64 dd (8.0, 2.0)	
4		49.8		49.8		47.7
5	1.37 dd (13.8, 8.4)	46.1	2.04 d (13.5)	55.4	1.83 d (13.5)	52.1
	1.93 dd (13.8, 9.0)		1.79 d (13.5)		1.97 d (13.5)	
6	2.47 m	49.4		88.0		72.7
7	2.36 dd (13.2, 8.4)	37.6	2.26 dd (13.0, 7.5)	48.3	2.33 dd (16.5, 8.0)	43.6
	1.53 dt (13.2, 6.0)		1.83 dd (13.0, 10.0)		1.91 dd (15.0, 8.0)	
8	4.82 br s	73.6	4.80 br s	75.1	4.79 br s	87.4
9		161.4		159.7		160.2
10		90.2	2.68 dd (7.0, 2.0)	58.3		89.7
11	2.04 s	65.4	1.62 m	69.1	1.88 s	67.0
12	5.40 d (1.8)	110.5	4.99 t (2.0)	107.8	5.38 d (2.0)	112.5
	5.43 d (1.8)		5.17 t (2.0)		5.42 d (2.0)	
13	1.16 s	32.7	1.21 s	32.1	1.31 s	33.4
14	1.17 s	27.0	1.04 s	30.0	1.03 s	31.8
15	3.61 d (11.4)	68.6	1.10 s	26.6	1.38 s	25.0
	3.89 d (11.4)					

^1^ Recorded in CDCl_3_.

Most capnellane sesquiterpenes possessed a terminal double bond Δ^9(12)^, which was characterized by two singlet proton peaks usually at *δ*_H_ 5.50–5.00 and 5.00–4.50 in the ^1^H NMR spectrum and two carbon signals appeared at *δ***_C_** ca. 160 and 110 ppm in the ^13^C NMR spectrum. In addition, hydroxylation frequently occurred for this type of sesquiterpenes. If it was hydroxylated at C-2, the ^1^H NMR signal at *δ*_H_ 4.03 ppm indicated the 2*β*-orientation for the hydroxyl group; whereas *δ*_H_ 3.57 ppm suggested the 2*α*-configuration. When the hydroxyl group was substituted at C-3, the proton resonated at *δ*_H_ 4.10 ppm. If the hydroxyl was on the 8*β* face, the ^1^H and ^13^C NMR chemical shifts of the methine were *δ*_H_ 4.80–4.74 ppm and *δ*_C_ 74.2–73.6 ppm, respectively; conversely, the ^1^H and ^13^C NMR data were *δ*_H_ 4.62 ppm and *δ*_C_ 72.0 ppm, respectively. However, the chemical shifts *δ*_H_ 4.51 ppm and *δ*_C_ 75.5 ppm of sesquiterpene **22** was an exception. Perhaps it was affected by other functional groups in its chemical structure. The occurrences of hydroxylation at C-5 and C-10 resulted in the carbon resonances at *δ*_C_ 82.8–81.4 ppm and *δ*_C_ 90.6–89.1 ppm, respectively. The chemical shifts of C-10 of compounds **4**, **17,** and **19** were inconsistent with the aforementioned rule, which revealed the possible influences caused by other substituents in their chemical structures.

#### 8.1.2. Precapnellane Sesquiterpene

Precapnelladiene (**30**) was the sole precapnellane sesquiterpene from soft corals of the genus *Capnella*. Only the ^1^H NMR data was recorded when it was discovered as a new hydrocarbon [69]. Until Paquette and co-workers completed the total synthesis of **30** [117], the ^13^C NMR data were reported.

Compound **30**: ^1^H NMR (CDCl_3_): *δ*_H_ 5.34 (t, *J* = 8.4 Hz, 1H), 5.06 (s, 1H), 3.54 (m, 1H), 2.91 (q, *J* = 11.5 Hz, 1H), 2.4 (m, 2H), 1.64 (s, 3H), 1.03 (d, *J* = 5.2 Hz, 3H), 0.97(s, 6H) [69]; ^1^H NMR (CDCI_3_): *δ*_H_ 5.33 (t, *J* = 8.3 Hz, 1H), 5.02 (d, *J* =1.5 Hz, 1H), 3.51 (dt, *J* = 13.0, 6.0 Hz, 1H), 2.90 (dd, *J* = 13.0, 9.3 Hz, 1H), 2.38 (m, 2H), 1.8–1.5 (br m, 4H), 1.63 (br s, 3H), 1.42 (m, 1H), 1.25 (m, 1H), 1.04 (d, *J* = 6.8 Hz, 3H), 0.99 (s, 3H), 0.97 (s, 3H) [117]; ^13^C NMR (CDCl_3_): *δ***_C_** 145.5, 136.2, 130.3, 121.8, 42.4, 40.5, 39.6, 38.9, 38.7, 33.7, 31.5, 31.3, 29.8, 26.7, 22.0 [117].

Compared to the common structural features of capnellane sesquiterpenes, the distinct characteristics of **30** were attributable to two isolated olefinic protons (*δ*_H_ 5.34 and 5.06 ppm), one allylic methyl (*δ*_H_ 1.64 ppm) and one doublet methyl (*δ*_H_ 1.03 ppm).

#### 8.1.3. Bicyclogermacrane Sesquiterpenes

All the ^1^H and ^13^C NMR data of capgermacrenes A–G (**31**–**37**) were available and supplemented in Table 8, Table 9 and Table 10.

The characteristic of these secondary metabolites was the presence of a cyclopropane ring. As demonstrated in Table 8, Table 9 and Table 10, the ^1^H NMR peaks at *δ*_H_ 1.45–0.82 and 1.39–0.68 ppm were two indicative signals of H-1 and H-10 of the cyclopropane in the chemical structures of **31**–**37**. Interestingly, if there was no exomethylene at C-3, one signal of the cyclopropane appeared at *δ*_H_ 0.77 or ca. 1.38 ppm, and the other appeared at *δ*_H_ 1.32 or ca. 1.44 ppm. When the exomethylene existed at C-3, two signals of the cyclopropane were observed at *δ*_H_ 1.03–0.82 ppm and *δ*_H_ 1.06–0.87 ppm. However, in the ^13^C NMR data of C-1 (*δ*_C_ 33.8–25.0 ppm) and C-2 (*δ*_C_ 30.1–24.7 ppm), it was it difficult to recognize the presence of cyclopropane, due to the confusing wide ranges of their chemical shifts.

Another common structural feature of these secondary metabolites was the α,β-conjugated ketone moiety. The chemical shift of H-6 could be regarded as an indicator of the configuration of double bond Δ^6^ in the subunit α,β-conjugated ketone. No matter the presence or absence of exomethylene at C-3, the ^1^H NMR signal resonating at *δ*_H_ > 6.0 ppm supported the *E*-configuration of Δ^6^, whereas *δ*_H_ < 6.0 ppm suggested the *Z*-configuration. For compounds **34**–**37**, the difference of ^13^C NMR data of C-6 and C-7 could be used to determine the configuration of Δ^6^. As illustrated in Table 9 and Table 10, the chemical-shift difference Δ*δ*_C_≈0 ppm was observed for the *E*-configuration of Δ^6^; conversely, the chemical-shift difference Δ*δ*_C_ > 6 ppm.

#### 8.1.4. Germacrane Sesquiterpene

Only compound capgermacrene H (**39**) was found as a new germacrane sesquiterpene. The ^1^H and ^13^C NMR data of this sesquiterpene were measured in two solvents CDCl_3_ and C_6_D_6_ (Table 11), respectively.

The major difference between compound **39** and the seven bicyclogermacrane sesquiterpenes **31**–**37** was the presence of the isopropenyl group instead of the cyclopropane. The ^1^H NMR signals at *δ*_H_ 4.79, 4.78, and 1.76 ppm recorded in CDCl_3,_ and ^13^C NMR peaks at *δ*_C_ 145.9, 110.3, and 20.9 ppm recorded in CDCl_3_ were attributable to the isopropenyl group.

#### 8.1.5. Farnesane Sesquiterpenes

The ^1^H and ^13^C NMR data of two farnesane sesquiterpenes **42** and **43** are shown in Table 12.

The chemical structures of these two compounds differed in the geometry of the double bond Δ^9^. As revealed in Table 12, the ^1^H NMR chemical shift of H-9 of the ***Z***-geometry of Δ^9^ in **42** moved upfield, compared with that of **43**. Meanwhile, the ^13^C NMR chemical shift of C-9 in **42** was upfield, too.

#### 8.1.6. Guaiane Sesquiterpene

The ^1^H and ^13^C NMR data of guaiane sesquiterpene **44** are listed in Table 13.

### 8.2. Diterpenes

Four diterpenes were reported from soft corals of the genus *Capnella*, which were xenicanes **45**–**48**. Their ^1^H and ^13^C NMR data are displayed in Table 14 and Table 15.

It is important to assign the *cis*/*trans*-fusion of two macro rings in the xenicane diterpenes. For compounds **47** and **48** that shared the same bicyclic nucleus, the characteristic chemical shifts of H-4a and H-11a were *δ*_H_ ca. 3.38 and 2.61 ppm, respectively, while the diagnostic chemical shifts of C-4a and C-11a were *δ*_C_ ca. 28.4 and 48.0 ppm, respectively (Table 15). However, these ^1^H and ^13^C NMR data changed with the variations of functional groups on the bicyclic nucleus. The remarkable difference in chemical shifts for diterpenes **45** and **46** is reported in Table 14.

### 8.3. Steroids

Among this group of secondary metabolites from soft corals of the genus *Capnella*, more than half were discovered as new compounds, whose ^1^H and ^13^C NMR data are provided in Table 17, Table 18, Table 19 and Table 20. Since ^1^H and ^13^C NMR data of some known compounds such as **50**, **51**, and **53** were recorded in the literature, their NMR data will be given in this manuscript (Table 16). It might be worth pointing out that the ^13^C NMR data of new compound **55** were not given in the literature. Additionally, the full assignments of the ^1^H and ^13^C NMR data of **54** as well as the ^1^H NMR data of **55** were not completed.

Compound **54**: ^1^H NMR (CDCl_3_): *δ*_H_ 6.81 (d, *J* = 8.5 Hz, 1H), 6.69 (d, *J* = 8.5 Hz, 1H), 5.80 (m, 1H), 5.66 (s, 1H), 4.98 (d, *J* = 14 Hz, 2H), 3.86 (s, 3H), 2.93 (dd, *J* = 17, 5 Hz, 1H), 2.64 (m, 1H), 2.31–1.17 (m, 14H), 0.62 (s, 3H) [74]; ^13^C NMR (CDCl_3_): *δ***_C_** 144.04, 142.94, 139.83, 134.56, 123.46, 115.95, 114.56, 107.96, 56.13, 55.58, 54,90, 44.32, 43.84, 38.50, 37.66, 27.41, 27.41, 26.43, 24.50, 23.40, 12.88 [74].

Compound **55**: ^1^H NMR (CDCl_3_): *δ*_H_ 7.12 (d, *J* = 9 Hz, 1H), 6.56 (dd, *J* = 9, 2 Hz, 1H), 6.51 (s, 1H), 5.8 (m, 1H), 4.93 (d, *J* = 14 Hz, 2H), 2.65 (m, 2H), 2.4–1.1 (m, 15H), 0.60 (s, 3H) [74].

Compound **56**: ^1^H NMR (CDCl_3_): *δ*_H_ 7.05 (d, *J* = 10 Hz, 1H), 5.75 (d, *J* = 10 Hz, 1H), 5.65 (m, 1H), 4.9 (d, *J* = 14 Hz, 2H), 2.4–1.1 (m, 19H), 1.0 (s, 3H), 0.60 (s, 3H) [74]; the later full assignments of ^1^H and ^13^C NMR (CDCl_3_): see Table 17.

The typical ^1^H NMR chemical shift at *δ*_H_ ca. 3.51 ppm was indicative of β-OH at C-3 of steroids. Usually, there was a double bond Δ^5^ in the chemical structures, which was supported by the characteristic ^1^H NMR chemical shift at *δ*_H_ ca. 5.30 ppm and the ^13^C NMR chemical shifts at *δ*_C_ ca. 141 and 121 ppm. For the vinyl group, its ^1^H and ^13^C NMR signals were usually observed at *δ*_H_ ca. 5.75 and 5.00 ppm and *δ*_C_ ca. 136.0 and 116.0 ppm. The diagnostic resonances at *δ*_H_ ca. 0.45 and –0.14 ppm in the ^1^H NMR spectrum along with *δ*_C_ ca. 21.5 ppm in the ^13^C NMR spectrum was the evidence of the subunit cyclopropane in the side chain.

### 8.4. Lipid

Although **69** was a previously reported lipid, its ^1^H and ^13^C NMR data were given in the literature.

Compound **69**: ^1^H NMR (CDCl_3_): *δ*_H_ 2.40 (t, *J* = 7.5 Hz, 2H), 2.12 (s, 3H), 1.58–1.52 (m, 2H), 1.29–1.22 (m, 26H)**,** 0.87 (s, 3H) [47]; ^13^C NMR (CDCl_3_): *δ*_C_ 209.43, 43.84, 31.92, 29.85, 29.68 (×11), 29.65, 29.60, 29.47, 29.35, 23.88, 22.69, 14.12 [47].

## 9. Progress on the Chemical Synthesis of Sesquiterpenes from Soft Corals of the Genus *Capnella*

Among the secondary metabolites from soft corals of the genus *Capnella*, the complex polycyclic features of sesquiterpenes made them attractive targets for synthetic chemists. Since the early 1980s, dozens of chemical synthetical works have been reported, which mainly focused on the sesquiterpenes belonging to three types: capnellane, precapnellane, and guaiane.

### 9.1. Capnellane Sesquiterpenes

#### 9.1.1. Δ^9(12)^-Capnellene (**6**)

This compound was the first chemically synthesized capnellane terpene as early as 1981 [118]. Since the beginning of the 1980s, a great number of chemists devoted themselves to the total synthesis of **6**, developing an array of different starting materials including 2,2,5-trimethyl-5-hexenoic acid [118,119], cyclopentenyl carboxaldehyde [120], 2,2,5-trimethyl-5-hexenal [121], methylcyclopentadiene isomers and *p*-benzoquinone [122], trimethylcyclopentanone [123], 2-cyclopentenone [124], vinyl lactone [125], *α*,*α*-dimethyl-*γ*-lactone [126], 1,3-cyclopentadiene [127], 8,8-dimethylbicyclo[3.3.0]oct-1(5)-en-2-one [128], 3-butyn-l-ol [129], bicyclic lactam [130], *α*,*α*-dimethyl-7-butyrolactone [131], ester-substituted fulvene [132], (+)-Δ^3^-carene [133], (-)-2-methyl-4-trimethylsilyl-2-cyclopen~en-1-one [134], bicyclo[3.3.0]octane derivative [135], oxodicyclopentadiene [136], *p*-cresol [137,138], substituted cyclopentanone [139], 2-methoxy-4-methylphenol [140], 2,2-dimethylpent-4-enal [55], and cyclopropanated cyclopentenone [56].

**Scheme 1:**
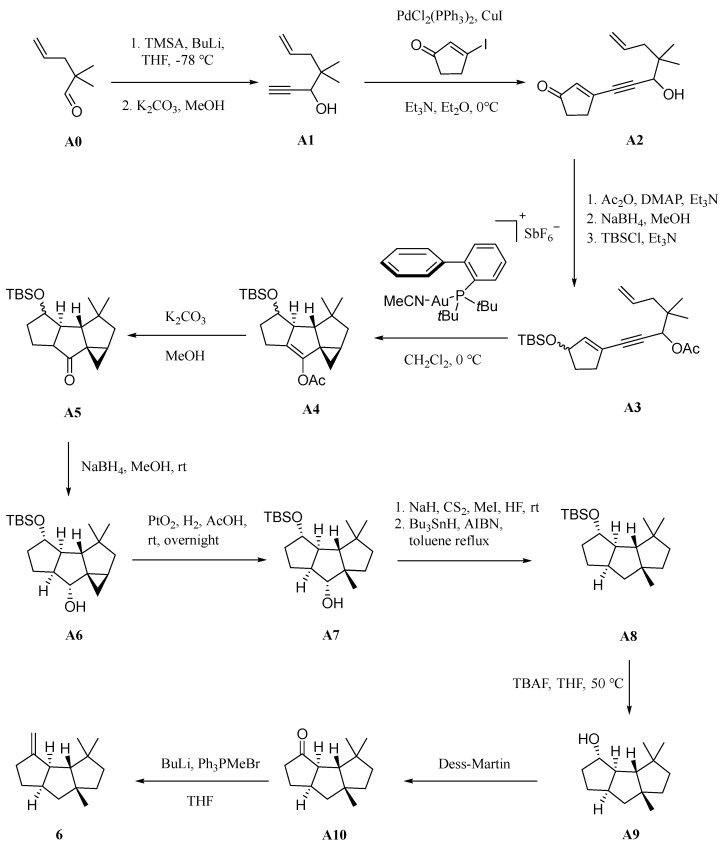
The route of total synthesis of **6** reported in the literature [55].

Malacria et al. completed total synthesis of Δ^9(12)^-capnellene (**6**) using 2,2-dimethylpent-4-enal **A0** as the starting material (Scheme 1) [55]. As outlined in Scheme 1, the first intermediate product **A1** coupled with 3-iodocyclopent-2-enone to yield enynol **A2**, which was the precursor of **A3**. Catalyzed by the gold complex, **A3** was transformed to triquinane **A4**. Undergoing a series of reactions, the capnellane **A7** was gained from **A4**. A series of transformations conducted on **A7** resulted in the key intermediate product **A10**. Finally, the methylenation of **A10** afforded the desired **6**.

**Scheme 2:**
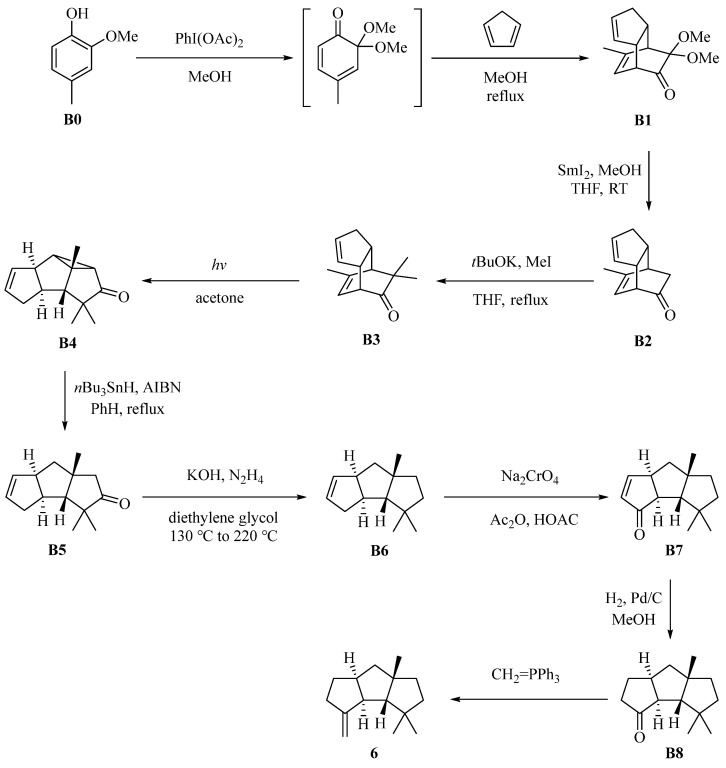
The route of total synthesis of **6** reported in the literature [140].

Hsu et al. accomplished the total synthesis of **6** from 2-methoxy-4-methylphenol **B0** (Scheme 2) [140]. As shown in Scheme 2, Diels–Alder reaction of **B0** with cyclopentadiene yielded a cycloadduct **B1**, which was demethoxylated to afford **B2**. This product reacted with MeI to install the geminal dimethyl groups in **B3**. After the opening of the cyclopropane ring, Huang-Minlon reduction, and allylic oxidation, the linear triquinane **B7** was produced. This key intermediate was subjected to hydrogenation and olefination to furnish the expected **6**.

**Scheme 3:**
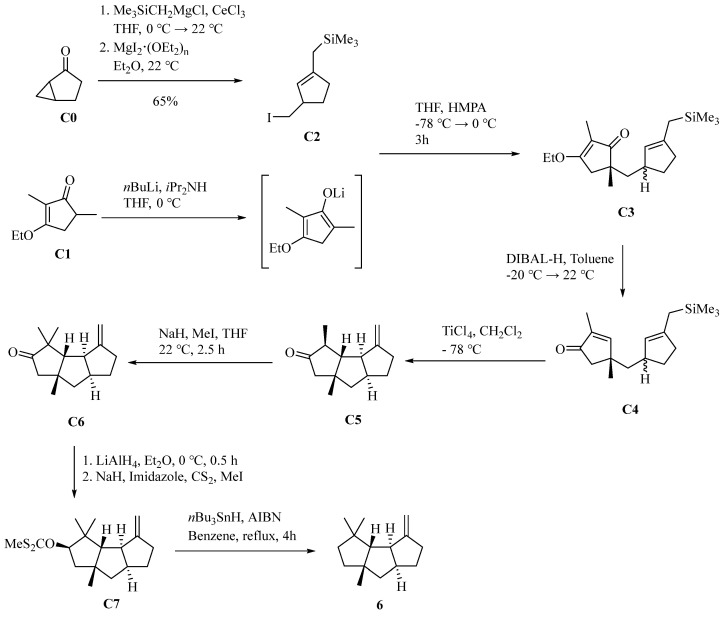
The route of total synthesis of **6** reported in the literature [56].

Li et al. accomplished the total synthesis of **6** from cyclopropanated cyclopentenone **C0** (Scheme 3) [56]. As depicted in Scheme 3, the condensation of **C1** and **C2**, which was the homoiodo allylsilane derivative of **C0**, yielded the bicyclic allylsilane **C3**. The expected tricyclic precursor **C5** was prepared from the reduction product **C4**, and subjected to the methylation to furnish the triquinone derivative **C6**. Following the standard Barton−McCombie procedure, the reductive deoxygenation of **C6** finally afforded **6**.

#### 9.1.2. Δ^9(12)^-Capnellene-3*β*,8*β*,10*α*-Triol (**1**)

In addition, the chemical synthesis of Δ^9(12)^-capnellene-3*β*,8*β*,10*α*-triol (**1**) was also investigated starting from 3-methyl-2-cyclopenten-1-one and 2-metyl-1,3-cyclopentanedione [54,141,142].

**Scheme 4:**
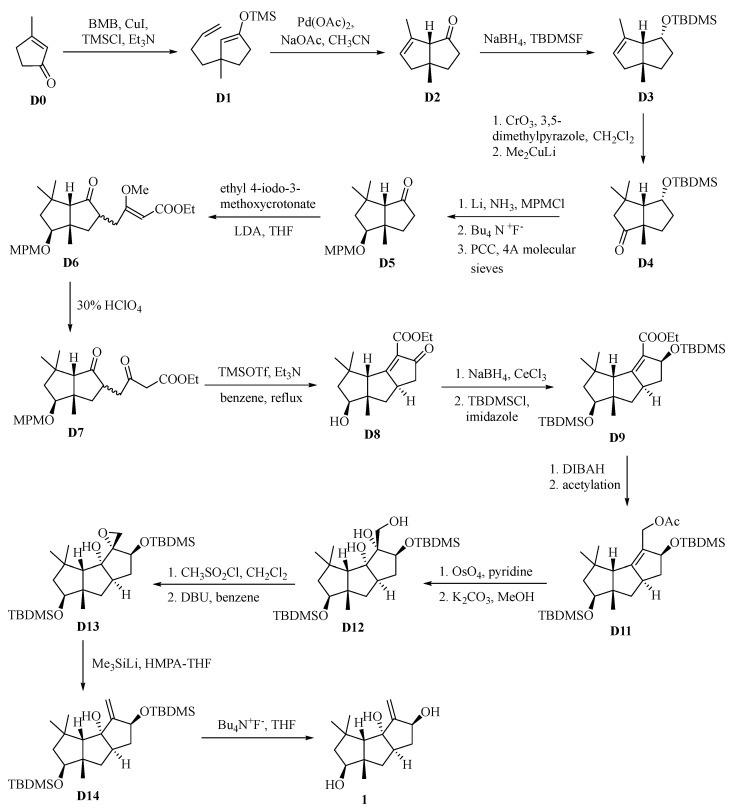
The route of total synthesis of **1** reported in the literature [54].

The first total synthesis of **1** starting from 3-methyl-2-cyclopenten-1-one **D0** was completed by Shibasaki et al. (Scheme 4) [54]. As displayed in Scheme 4, the initial product **D1** was obtained from the addition to **D0**, which was the precursor of the cyclized product of **D2**. **D4** was yielded from **D2** after undergoing reduction, allylic oxidation, and methylation. The sequential reduction, protection, desilylation, and oxidation afforded an important derivative **D5**. After a series of reactions, the key tricyclic intermediate **D8** was obtained. Reduction and silylation of **D8** afforded **D9**, which was further reduced to yield **D11**. The following oxidation, epoxidation, elimination, and deprotection produced the desired **1**.

#### 9.1.3. Δ^9(12)^-Capnellene-8*β*,10*α*-Diol (**2**) and Its 8-Epimer Δ^9(12)^-Capnellene-8*α*,10*α*-Diol (**70**)

The efforts on the chemical synthesis of Δ^9(12)^-capnellene-8*β*,10*α*-diol (**2**) from 3-methyl-2-cyclopenten-1-one [54], enediol [143], and 3-methylcyclopent-2-enone [144] were reported.

**Scheme 5:**
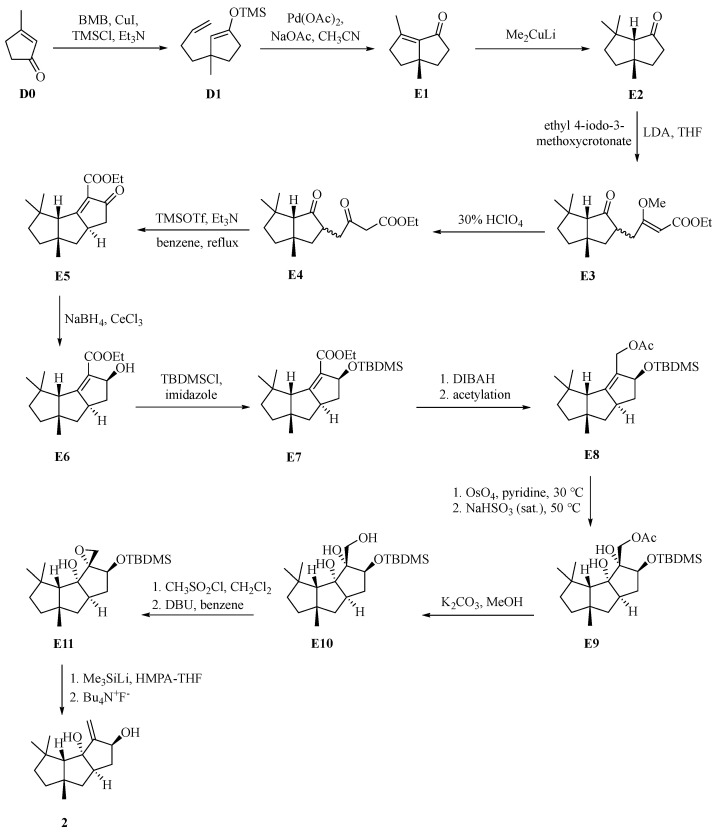
The route of total synthesis of **2** reported in the literature [54].

Shibasaki et al. also reported the first total synthesis of **2** starting from 3-methyl-2-cyclopenten-1-one **D0** (Scheme 5) [54]. As displayed in Scheme 5, the aforementioned product **D1** could be transformed to **E1**, which yielded **E2** after methylation. This intermediate product was subjected to a series of reactions to afford the key tricyclic intermediate **E5**. Reduction of **E5** gave **E6**, which was silylated to produce **E7**. Followed by reduction, epoxidation, elimination, and deprotection, **2** was successfully yielded.

**Scheme 6:**
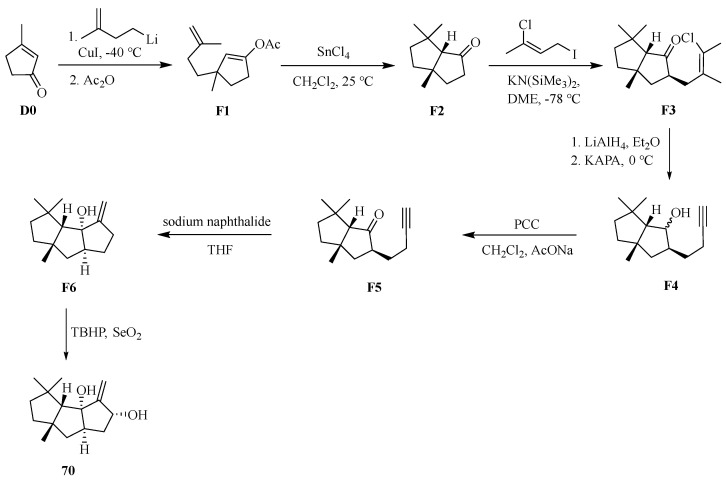
The route of total synthesis of **70** reported in the literature [53].

3-Methylcyclopent-2-enone **D0** can be used as the starting material to synthesize the 8-epimer of **2**, namely Δ^9(12)^-capnellene-8*α*,10*α*-diol (**70**) (Scheme 6), which was reported by Pattenden et al. [53]. As shown in Scheme 6, alkylation of **D0** followed by quenching with acetic anhydride led to the enol acetate **F1**, which was further converted to bicyclooctanone **F2**. Alkylation of **F2** afforded the keto-olefin **F3**. The following reduction and oxidation yielded the key product **F5**. Cyclisation of **F5** furnished tricyclic derivative **F6**. Treatment of **F6** with THBP in the presence of catalytic SeO_2_ gave **70**.

#### 9.1.4. Δ^9(12)^-Capnellene-3*β*,8*β*,10*α*,14-Tetrol (**3**)

The chemical synthesis of Δ^9(12)^-capnellene-3*β*,8*β*,10*α*,14-tetrol (**3**) have also been conducted, using 2-methyl-1,3-pentanedione [141], 3-methylcyclopent-2-enone [142] and its derived ketone [145] as starting materials. 

**Scheme 7:**
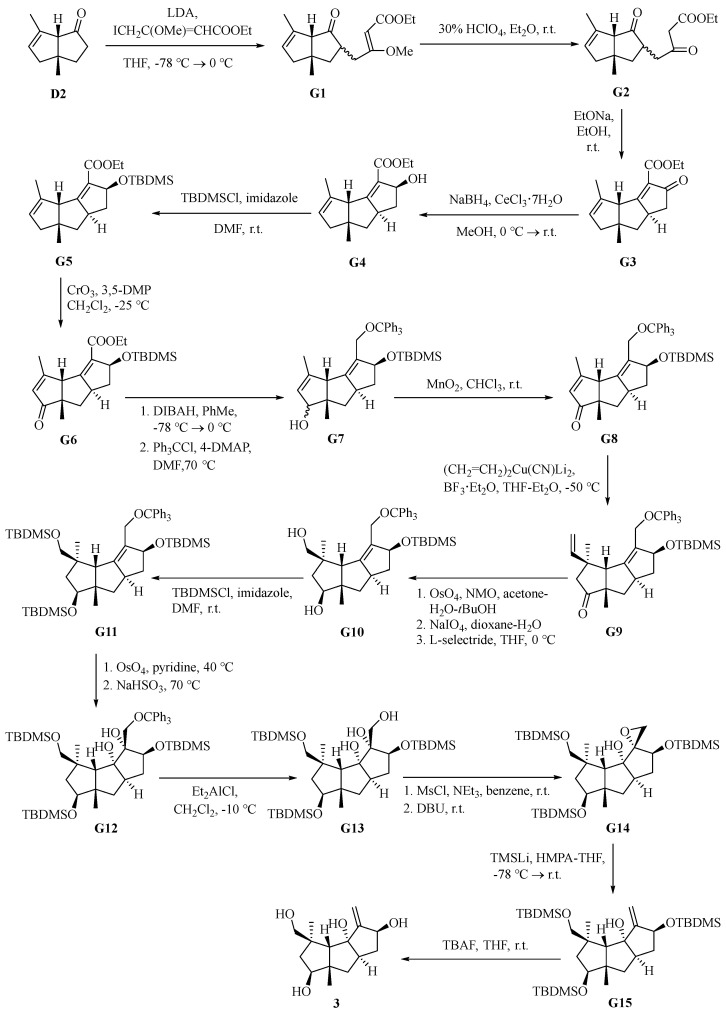
The route of total synthesis of **3** reported in the literature [145].

Shibasaki et al. also reported the first total synthesis of **3** starting from the above-mentioned ketone **D2** (Scheme 7) [145]. As depicted in Scheme 7, **G1** underwent alkylation, oxidation, and addition, affording the tricyclic derivative **G3**. After an array of reactions including oxidation, silylation, and reduction, **G3** was converted to the key ketone **G8**. The following conjugate addition of a vinyl group furnished **G9**, which could transfer to the hydroxymethyl product **G10**. The subsequent silylation, oxidation, epoxidation, elimination, and desilylation yielded the expected sesquiterpene **3**.

### 9.2. Precapnellane Sesquiterpene

#### 9.2.1. Precapnelladiene (**30**)

The hydrocarbon precapnelladiene (**30**) was considered as a possible biosynthetic precursor to various tricyclo[6.3.0.0^2,6^]-undecanes such as capnellane sesquiterpene **2**. Due to the intriguing structural features, the total synthesis has been extensively studied, in which tricyclic bis-enone [146], 6-alkenyl-2-methylenetetrahydropyran [147], 8*α*-methylbicyclo[3.3.0]octan-2-one [117], cyclopentapentalenedione [148], 3-methylcyclopentenone [149], 4,4-dimethyl-2-cyclohexen-1-one [150], ethyl 2-oxocyclopentanecarboxylate [151], diisopropylsquarate [152,153], and 2-methoxycarbonyl-2-cyclopenten-1-one [154] served as the starting materials.

**Scheme 8:**
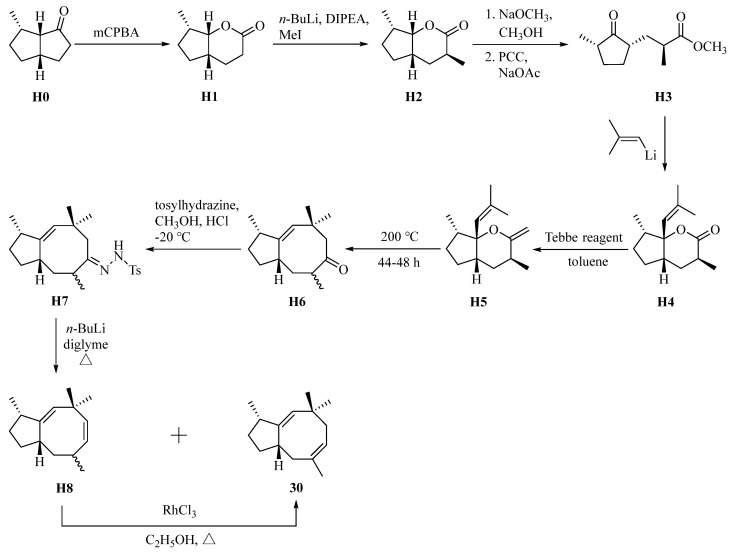
The route of total synthesis of **30** reported in the literature [117].

Paquette et al. completed the total synthesis of **30** from 8*α*-methylbicyclo[3.3.0]octan-2-one **H0** (Scheme 8) [117]. As outlined in Scheme 8, Baeyer-Villiger oxidation of **H0** provided **H1**, which was methylated to give **H2**. Cleavage oxidation of **H2** produced **H3**, which was the precursor of **H4**. Treatment of **H4** by Tebbe reagent followed by thermolysis afforded precapnellane derivative **H6**. The tosylhydrazone **H7** was decomposed to give a mixture of the desired **30** and the isomer **H8**. Additionally, RhCl_3_-promoted isomerization of **H8** yielded **30**.

**Scheme 9:**
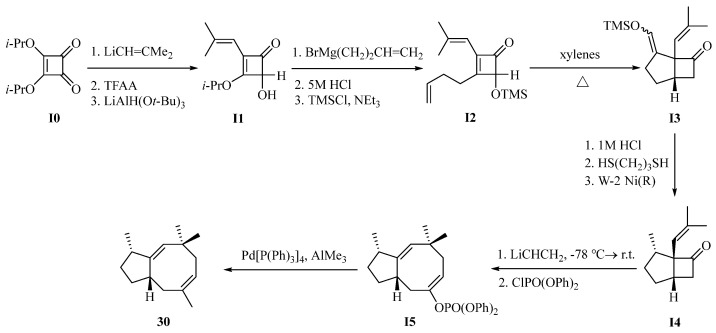
The route of total synthesis of **30** reported in the literature [152].

Moore et al. completed a total synthesis of **30** from diisopropylsquarate **I0** (Scheme 9) [152]. As depicted in Scheme 9, **I0** was subjected to addition, trifluoroacetylation, and chemoselective reduction, leading to alcohol **I1**. This alcohol was treated by a Grignard reagent, followed by hydrolysis and trimethylsilylation to give **I2**. Heating **I2** yielded bicyclic derivative **I3**. The subsequent hydrolysis, thioacetalization, and reduction of **I3** produced ketone **I4**. The oxy-Cope rearrangement provided the key precapnellane derivative **I5**. Finally, dephosphorization of **I5** led to the desired **30**.

**Scheme 10:**
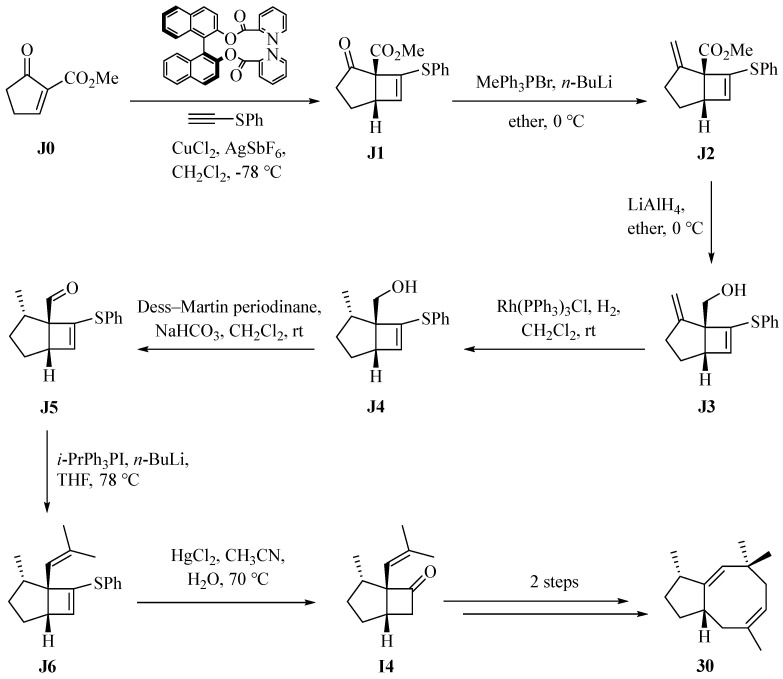
The route of total synthesis of **30** reported in the literature [154].

Iguchi et al. synthetized **30** from 2-methoxycarbonyl-2-cyclopenten-1-one **J0** (Scheme 10) [154]. As shown in Scheme 10, cycloaddition to **J0** yielded the bicyclic derivative **J1**, which was converted to **J2** by the Witting reaction. The subsequent reduction and oxidation of **J2** afforded the aldehyde **J5**, which was transformed to **J6** by the Witting reaction. Hydrolysis of **J6** provided the key intermediate product **I4**. Conversion of **I4** to the expected **30** was achieved according to the aforementioned procedure [152].

#### 9.2.2. Epiprecapnelladiene (**71**)

Meanwhile, the epimer of natural product **30** called epiprecapnelladiene (**71**) was synthesized [155,156].

**Scheme 11:**
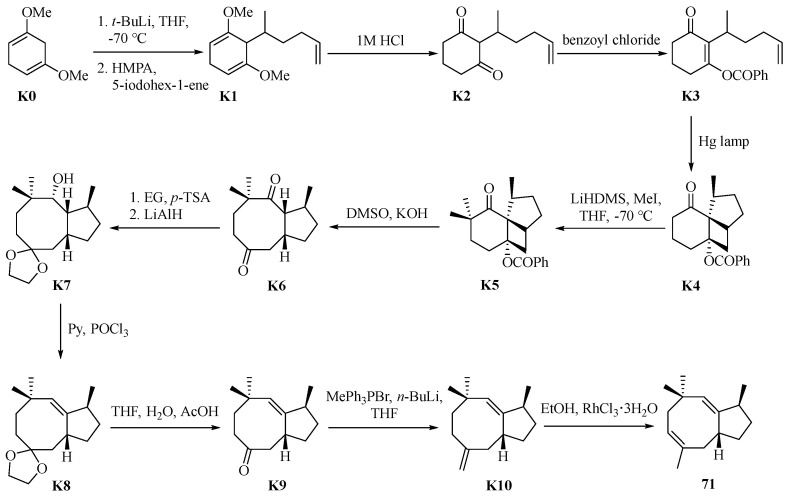
The route of total synthesis of **71** reported in the literature [156].

Pattenden et al. synthesized **71** from 2,4-dimethoxycyclohexa-1,4-diene **K0** (Scheme 11) [156]. As displayed in Scheme 11, alkylation of **K0** afforded the bisether **K1**, which was subjected to hydrolysis and benzoylation to give **K3**. Methylation of the tricyclic photoadduct **K4** afforded **K5**. Ring cleavage of **K5** yielded the key bicyclic intermediate product **K6**. This compound was converted to **K9** after a series of reactions including aldol condensation, reduction, elimination, and hydrolysis. The Witting reaction of **K9** produced **K10**, which transformed to **71** by isomerization.

### 9.3. Guaiane Sesquiterpene

#### Oxyfungiformin (**44**)

**Scheme 12:**
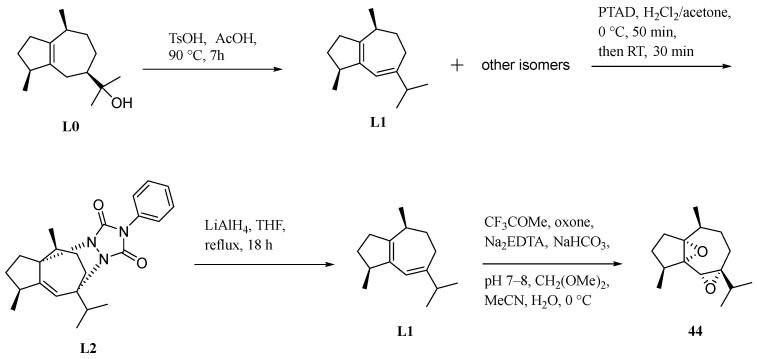
The route of total synthesis of **44** reported in the literature [97].

Chemical scientists were interested in the one guaiane sesquiterpene, oxyfungiformin (**44**), obtained from soft corals of the genus *Capnella*, and carried out the synthesis (Scheme 12) [97].

The selected starting material was guaiol **L0**, from which a mixture of *γ*-guaiene **L1** and its diene isomers were obtained after dihydroxylation. To get the enantiomerically pure **L1**, the Diels–Alder addition and the decomposition of the product **L2** were conducted. Lastly, double epoxidation of **L1** yielded the desired oxyfungiformin (**44**) [97].

## 10. Conclusions

As presented in this work, 69 secondary metabolites were summarized from soft corals of the genus *Capnella* over the five decades (Table 1). Based on the general structural features, these chemical constituents can be grouped as sesquiterpenes, diterpenes, steroids, and lipids. Additionally, the ^1^H and ^13^C NMR data of these metabolites were provided when available in the literature. Interestingly, these components displayed a variety of pharmacological activities including cytotoxic, antibacterial, antifungal, anti-inflammatory, and tyrosine kinase inhibitory activities (Table 1). Due to the intriguing structural features and significant bioactivities, chemical scientists developed a vast library of strategies for the synthesis of capnellanes **1**, **2**, **3**, and **6**, precapnellane **30,** and guaiane **44**. Moreover, the epimers of **2** and **30** were synthesized.

As displayed on the website of the Word Register of Marine Species (WoRMS) [157], there are 29 species in the genus *Capnella*. However, less than one-third of the species have been chemically investigated till now (Figure 2). The limited amounts of studied species afforded a variety of secondary metabolites, which underlined the demand for a more in-depth exploration of this genus. Furthermore, it is likely that the chemotaxonomic significance of secondary metabolites needs to be highlighted, which could support the revisionary systematics of Octocorallia [43]

It might be worth pointing out that genome mining was recently applied to identify capnellane synthase CiTC-1 from *C. imbricata*, which could produce secondary metabolites **2** and **6** [158]. This would inspire more chemists to devote themselves to exploring more *Capnella* soft corals-encoded terpene cyclases.

## Figures and Tables

**Table 1:** Secondary metabolites from soft corals of the genus *Capnella* from 1974 to May 2024.

No.	Name	Class	Species	Locality	Bioassays	Ref.
**1**	Δ^9(12)^-capnellene-3*β*,8*β*,10*α*-triol	capnellane sesquiterpene	*Capnella imbricata*	Leti Island, Indonesia	– ^1^	[52]
			*C. imbricata*	Leti Island, Indonesia	– ^1^	[57]
			*C. imbricata*	Leti Island, Indonesia	– ^1^	[58]
**2**	Δ^9(12)^-capnellene-8*β*,10*α*-diol	capnellane sesquiterpene	*C. imbricata*	Leti Island, Indonesia	– ^1^	[58]
			*C. imbricata*	Lakor Island, Indonesia	– ^1^	[58]
			*C. imbricata*	Mayu Island, Indonesia	cytotoxic against cell lines HL-60, K562, G402, MCF-7, HT115, and A2780 (IC_50_ 51, 0.7, 42–51, 93, 63, and 9.7 μM, respectively)	[48]
			*C. imbricata*	Green Island, China	significantly reduced the levels of iNOS and COX-2 proteins (1.2 ± 0.1% and 24.8 ± 7.5%, respectively) at a concentration of 10 µM	[51]
			*C. imbricata*	Green Island, China	anti-neuroinflammatory and anti-nociceptive properties in IFN-γ-stimulated microglial cells and in neuropathic rats, respectively	[59]
			*C. imbricata*	Green Island, China	impaired vascular development in zebrafish	[60]
			*Capnella* sp.	Green Island, China	cytotoxic against cell lines Hela and KB (IC_50_ 3.56 and 6.06 μg/mL)	[61]
			*C. imbricata*	Orchid Island, China	Inactive against elastase release and superoxide generation by human neutrophils at a concentration of 10 μM	[62]
**3**	Δ^9(12)^-capnellene-3*β*,8*β*,10*α*,14-tetrol	capnellane sesquiterpene	*C. imbricata*	Leti Island, Indonesia	– ^1^	[63]
**4**	Δ^9(12)^-capnellene-5*α*,8*β*,10*α*-triol	capnellane sesquiterpene	*C. imbricata*	Lakor Island, Indonesia	– ^1^	[58]
			*C. imbricata*	Orchid Island, China	Inactive against elastase release and superoxide generation by human neutrophils at a concentration of 10 μM	[62]
			*C. imbricata*	Orchid Island, China	significant inhibition against COX-2 protein expression at a concentration of 10 µM	[44]
**5**	Δ^9(12)^-capnellene-2*β*,8*β*,10*α*-triol	capnellane sesquiterpene	*C. imbricata*	Lakor Island, Indonesia	– ^1^	[58]
			*C. imbricata*	Orchid Island, China	no obvious inhibition against COX-2 and iNOS expression at a concentration of 10 µM	[44]
**6**	Δ^9(12)^-capnellene	capnellane sesquiterpene	*C. imbricata*	Indonesia	– ^1^	[64]
**7**	Δ^9(12)^-capnellene-2*β*,5*α*,8*β*,10*α*-tetrol	capnellane sesquiterpene	*C. imbricata*	Laing Island, Papua New Guinea	– ^1^	[65]
**8**	8*β*-acetoxy-Δ^9(12)^-capnellene-10*α*-ol	capnellane sesquiterpene	*C. imbricata*	Laing Island, Papua New Guinea	– ^1^	[66]
**9**	3*β*-acetoxy-Δ^9(12)^-capnellene-8*β*,10*α*-diol	capnellane sesquiterpene	*C. imbricata*	Laing Island, Papua New Guinea	– ^1^	[66]
**10**	3*β*,8*β*-diacetoxy-Δ^9(12)^-capnellene-10*α*-ol	capnellane sesquiterpene	*C. imbricata*	Laing Island, Papua New Guinea	– ^1^	[66]
**11**	5*α*-acetoxy-Δ^9(12)^-capnellene-8*β*,10*α*-diol	capnellane sesquiterpene	*C. imbricata*	Laing Island, Papua New Guinea	– ^1^	[66]
**12**	5*α*,8*β*-diacetoxy-Δ^9(12)^-capnellene-10*α*-ol	capnellane sesquiterpene	*C. imbricata*	Laing Island, Papua New Guinea	– ^1^	[66]
**13**	3*β*,14-diacetoxy-Δ^9(12)^-capnellene-8*β*,10*α*-diol	capnellane sesquiterpene	*C. imbricata*	Laing Island, Papua New Guinea	– ^1^	[66]
**14**	2*β*,5*α*-diacetoxy-Δ^9(12)^-capnellene-8*β*,10*α*-diol	capnellane sesquiterpene	*C. imbricata*	Laing Island, Papua New Guinea	– ^1^	[66]
**15**	2*β*,5*α*,8*β*-triacetoxy-Δ^9(12)^-capnellene-10*α*-ol	capnellane sesquiterpene	*C. imbricata*	Laing Island, Papua New Guinea	– ^1^	[66]
**16**	Δ^9(12)^-capnellene-8*α*,10*β*-diol	capnellane sesquiterpene	*C. imbricata*	Xisha Islands, China	– ^1^	[67]
**17**	Δ^9(12)^-capnellene-2*α*,5*β*,8*α*,10*β*-tetrol	capnellane sesquiterpene	*C. imbricata*	Xisha Islands, China	suppressive action on contracture of the removed ileums of the guinea pig and antitumor effects on EAC at 25 μg/mL with an inhibition rate of 43%	[68]
**18**	Δ^9(12)^-capnellene-8*β*-ol	capnellane sesquiterpene	*C. imbricata*	Mayu Island, Indonesia	cytotoxic against cell lines HL-60, K562 and A2780 (IC_50_ 68, 4.6 and 6.6 μM, respectively)	[48]
			*C. imbricata*	Green Island, China	inactive in the bioassays	[51]
			*C. imbricata*	Orchid Island, China	no obvious inhibition against COX-2 and iNOS expression at a concentration of 10 µM	[44]
			*C. imbricata*	Green Island, China	anti-neuroinflammatory and anti-nociceptive properties in IFN-γ-stimulated microglial cells and in neuropathic rats, respectively	[59]
**19**	3*β*-acetoxy-Δ^9(12)^-capnellene-8*β*,10*α*,14*β*-triol	capnellane sesquiterpene	*C. imbricata*	Mayu Island, Indonesia	cytotoxic against cell lines HL-60, K562, G402, MCF-7, and A2780 (IC_50_ 713, 24, 52, 1029, and 32 μM, respectively)	[48]
**20**	8*α*-acetoxy-Δ^9(12)^-capnellene-10*α*-ol	capnellane sesquiterpene	*C. imbricata*	Green Island, China	significantly reduced the levels of iNOS and COX-2 proteins (54.4 ± 12.0% and 62.9 ± 13.7%, respectively) at a concentration of 10 µM	[51]
			*C. imbricata*	Orchid Island, China	significant inhibition against iNOS and COX-2 protein expressions at a concentration of 10 µM	[44]
**21**	Δ^9(12)^-capnellene-10*α*-ol-8-one	capnellane sesquiterpene	*C. imbricata*	Green Island, China	significantly reduced the levels of iNOS protein (34.8 ± 10.2%) at a concentration of 10 µM	[51]
**22**	Δ^9(12)^-capnellene-8*β*,15-diol	capnellane sesquiterpene	*C. imbricata*	Green Island, China	inactive in the bioassays	[51]
**23**	Δ^9(12)^-capnellene-8*β*,10*α*,13- triol	capnellane sesquiterpene	*C. imbricata*	Green Island, China	inactive in the bioassays	[51]
**24**	Δ^9(10)^-capnellene-12-ol-8-one	capnellane sesquiterpene	*C. imbricata*	Green Island, China	inactive in the bioassays	[51]
**25**	8*β*,10*α*-diacetoxy-Δ^9(12)^-capnellene	capnellane sesquiterpene	*C. imbricata*	Green Island, China	inactive in the bioassays	[51]
**26**	8*β*-acetoxy-Δ^9(12)^-capnellene	capnellane sesquiterpene	*C. imbricata*	Green Island, China	inactive in the bioassays	[51]
**27**	Δ^9(12)^-capnellene-8*β*,10*α*,15-triol	capnellane sesquiterpene	*C. imbricata*	Orchid Island, China	significant inhibitory effects on elastase release and superoxide generation by human neutrophils with inhibition rate of 5.67% and 9.28%, respectively	[62]
			*C. imbricata*	Orchid Island, China	no obvious inhibition against COX-2 and iNOS expression at a concentration of 10 µM	[44]
**28**	Δ^9(12)^-capnellene-6*α*,8*β*-diol	capnellane sesquiterpene	*C. imbricata*	Orchid Island, China	significant inhibition against COX-2 protein expression (12.57%) at a concentration of 10 µM	[44]
**29**	Δ^9(12)^-capnellene-6*α*,8*β*,10*α*-triol	capnellane sesquiterpene	*C. imbricata*	Orchid Island, China	remarkable decrease in iNOS level (27.73%) at a concentration of 10 µM	[44]
**30**	precapnelladiene	precapnellene sesquiterpene	*C. imbricata*	Indonesia	– ^1^	[69]
**31**	capgermacrene A	bicyclogermacrane sesquiterpene	*Capnella* sp.	Mantanani Island, Malaysia	inhibited the accumulation of the LPS-induced pro-inflammatory IL-1β and NO production by down-regulating the expression of iNOS protein in RAW 264.7 macrophages	[70]
			*C. imbricata*	Mantanani Island, Malaysia	cytotoxic against S1T cells (IC_50_ = 0.79 μg/mL)	[71]
**32**	capgermacrene B	bicyclogermacrane sesquiterpene	*Capnella* sp.	Mantanani Island, Malaysia	inactive in the bioassays	[70]
			*C. imbricata*	Mantanani Island, Malaysia	cytotoxic against S1T cells (IC_50_ = 7.79 μg/mL)	[71]
**33**	capgermacrene C	bicyclogermacrane sesquiterpene	*Capnella* sp.	Mantanani Island, Malaysia	negligible inhibition against *Escherichia coli* and *Staphylococcus aureus* (MIC > 500 μg/mL)	[72]
			*C. imbricata*	Mantanani Island, Malaysia	– ^1^	[71]
**34**	capgermacrene D	bicyclogermacrane sesquiterpene	*C. imbricata*	Mantanani Island, Malaysia	bacteriostatic activity against *S*. *aureus* and MRSA	[49]
			*C. imbricata*	Mantanani Island, Malaysia	cytotoxic against S1T cells (IC_50_ = 6.19 μg/mL)	[71]
**35**	capgermacrene E	bicyclogermacrane sesquiterpene	*C. imbricata*	Mantanani Island, Malaysia	bacteriostatic activity against *S*. *aureus* and MRSA	[49]
			*C. imbricata*	Mantanani Island, Malaysia	cytotoxic against S1T cells (IC_50_ = 4.75 μg/mL)	[71]
**36**	capgermacrene F	bicyclogermacrane sesquiterpene	*C. imbricata*	Mantanani Island, Malaysia	bactericidal activity against *S*. *aureus* and MRSA	[49]
			*C. imbricata*	Mantanani Island, Malaysia	cytotoxic against S1T cells (IC_50_ = 2.39 μg/mL)	[71]
**37**	capgermacrene G	bicyclogermacrane sesquiterpene	*C. imbricata*	Mantanani Island, Malaysia	bactericidal activity against *S*. *aureus* and MRSA	[49]
			*C. imbricata*	Mantanani Island, Malaysia	cytotoxic against S1T cells (IC_50_ = 3.97 μg/mL)	[71]
**38**	litseagermacrane	germacrane sesquiterpene	*Capnella* sp.	Mantanani Island, Malaysia	inactive in the bioassays	[70]
**39**	capgermacrene H	germacrane sesquiterpene	*C. imbricata*	Mantanani Island, Malaysia	inactive against S1T cells (IC_50_ > 30.0 μg/mL)	[71]
**40**	palustrol	aromadendrane sesquiterpene	*Capnella* sp.	Mantanani Island, Malaysia	inactive in the bioassays	[70]
**41**	1,4-peroxy-5-muurolene	cadinane sesquiterpene	*Capnella* sp.	Mantanani Island, Malaysia	negligible inhibition against *E. coli* and *S. aureus* (MIC > 500 μg/mL)	[72]
**42**	ethyl 5-[(1*E*,5*Z*)-2,6 dimthlocta-1,5,7-trienyl]furan-3-carboxylate	farnesane sesquiterpene	*Capnella fungiformis*	Mahambo, Madagascar	– ^1^	[47]
**43**	ethyl 5-[(1*E*,5*E*)-2,6 dimthlocta-1,5,7-trienyl]furan-3-carboxylate	farnesane sesquiterpene	*C. fungiformis*	Mahambo, Madagascar	– ^1^	[47]
**44**	oxyfungiformin	guaiane sesquiterpene	*C. fungiformis*	Mahambo, Madagascar	– ^1^	[47]
**45**	tsitsixenicin A	xenicane diterpene	*Capnella thyrsoidea*	Tsitsikamma Marine Reserve, South Africa	good inhibition (>80%) of superoxide production in rabbit neutrophils at a concentration of 12.5 μg/mL; good inhibition (68%) of superoxide production in human neutrophils at a concentration of 1.25 μg/mL	[45]
**46**	tsitsixenicin B	xenicane diterpene	*C. thyrsoidea*	Tsitsikamma Marine Reserve, South Africa	inactive in the bioassays	[45]
**47**	tsitsixenicin C	xenicane diterpene	*C. thyrsoidea*	Tsitsikamma Marine Reserve, South Africa	good inhibition (>80%) of superoxide production in rabbit neutrophils at a concentration of 12.5 μg/mL	[45]
**48**	tsitsixenicin D	xenicane diterpene	*C. thyrsoidea*	Tsitsikamma Marine Reserve, South Africa	good inhibition (>80%) of superoxide production in rabbit neutrophils at a concentration of 12.5 μg/mL; moderate inhibition (21%) of superoxide production in human neutrophils at a concentration of 1.25 μg/mL	[45]
**49**	cholesterol	steroid	*C. imbricata*	Lesser Sunda, Indonesia	– ^1^	[73]
**50**	24-methylenecholesterol	steroid	*C. imbricata*	Lesser Sunda, Indonesia	– ^1^	[73]
			*C. fungiformis*	Mahambo, Madagascar	– ^1^	[47]
**51**	24-methylcholesterol	steroid	*C. imbricata*	Lesser Sunda, Indonesia	– ^1^	[73]
			*C. fungiformis*	Mahambo, Madagascar	– ^1^	[47]
**52**	*β*-sitosterol	steroid	*C. imbricata*	Lesser Sunda, Indonesia	– ^1^	[73]
**53**	gorgosterol	steroid	*C. imbricata*	Lesser Sunda, Indonesia	– ^1^	[73]
			*C. fungiformis*	Mahambo, Madagascar	– ^1^	[47]
**54**	3-methoxy-19-norpregna-1,3,5(10),20-tetraen-4-ol	steroid	*Capnella* sp. nov.	Tasmania, Australia	– ^1^	[74]
**55**	19-norpregna-1,3,5(10),20-tetraen-3-ol	steroid	*Capnella* sp. nov.	Tasmania, Australia	– ^1^	[74]
**56**	5*α*-pregna-1,20-dien-3-one	steroid	*Capnella erecta*	Tasmania, Australia	– ^1^	[74]
			*C. thyrsoidea*	Tsitsikamma Marine Reserve, South Africa	stimulated superoxide production in rabbit neutrophils	[45]
			*C. thyrsoidea*	Tsitsikamma Marine Reserve, South Africa	inhibitory activity against plasmodial proteases and selectivity on human cathepsins	[75]
**57**	16*β*-hydroxy-5*α*-pregna-l,20-dien-3-one 16-acetate	steroid	*C.* *thyrsoidea*	Tsitsikamma Marine Reserve, South Africa	stimulated superoxide production in rabbit neutrophils	[45]
**58**	3*α*,16*β*-dthydroxy-5α-pregna-1,20-diene 3,16-diacetate	steroid	*C.* *thyrsoidea*	Tsitsikamma Marine Reserve, South Africa	– ^1^	[45]
**59**	12*β*-acetoxy-7*α*-hydroxygorgosterol	steroid	*Capnella lacertiliensi*	Great Barrier Reef, Australia	antifungal against *Microbotryum violacea* and *Eurotium repens* (1 mm zone at a concentration of 20 µg and 2 mm zone at a concentration of 20 µg, respectively)	[50]
**60**	12*β*-acetoxy-7*α*,19-dihydroxygorgosterol	steroid	*C. lacertiliensi*	Great Barrier Reef, Australia	antifungal against *M*. *violacea* (2 mm zone at a concentration of 10 µg)	[50]
**61**	12*β*-acetoxyergost-5-ene-3*β*,23-diol	steroid	*C. lacertiliensi*	Great Barrier Reef, Australia	antifungal against *M*. *violacea* (3 mm zone at a concentration of 10 µg), inhibitory against the enzyme tyrosine kinase p56*^lck^* 42% at a concentration of 200 µg/ mL	[50]
**62**	12*β*-acetoxyergost-5-ene-3*β*,11*β*,16-triol	steroid	*C. lacertiliensi*	Great Barrier Reef, Australia	antifungal against *M. violacea* (1 mm zone at a concentration of 25 µg)	[50]
**63**	11*β*-acetoxyergost-5-ene-3*β*,12*β*,16-triol	steroid	*C. lacertiliensi*	Great Barrier Reef, Australia	antifungal against *M*. *violacea* and *E*. *repens* (3 mm zone at a concentration of 10 µg and 2 mm zone at a concentration of 10 µg, respectively), inhibitory against the enzyme tyrosine kinase p56*^lck^* 47% at a concentration of 200 µg/ mL	[50]
**64**	capnesterone A	steroid	*C*. *imbricata*	Orchid Island, China	inhibition against iNOS level (60%) and promotion against COX-2 release (134%)	[46]
**65**	capnesterone B	steroid	*C*. *imbricata*	Orchid Island, China	inhibition against iNOS level (82%) and promotion against COX-2 release (110%)	[46]
**66**	4*β*-hydroxy-24-methylene-5-cholesten-7-one	steroid	*C*. *imbricata*	Orchid Island, China	moderate reduction in iNOS level (64.409%) at a concentration of 10 μM	[76]
**67**	3*β*-hydroxy-24-methylene-5-cholesten-7-one	steroid	*C*. *imbricata*	Orchid Island, China	moderate reduction in iNOS level (77.200%) at a concentration of 10 μM	[76]
**68**	gorgostan-5,25-dien-3*β*-ol	steroid	*C*. *imbricata*	Orchid Island, China	moderate reduction in iNOS level (73.820%) at a concentration of 10 μM	[76]
**69**	octadecan-2-one	lipid	*C. fungiformis*	Mahambo, Madagascar	– ^1^	[47]

^1^ The sign ‘–’ indicated no bioassay for this compound was recorded in the work.

**Table 8:** The ^1^H and ^13^C NMR data of compounds **31**–**33**.

No.	31 [70]		32 [70]		33 [72]	
	*δ*_H_ ^1^	*δ*_C_ ^1^	*δ*_H_ ^1^	*δ*_C_ ^1^	*δ*_H_ ^1^	*δ*_C_ ^1^
1	1.32 dd (10.3, 8.9)	26.1	1.45 t (8.9)	27.9	1.44 dd (9.6, 9.6)	25.0
2	4.87 d (10.3)	125.2	4.61 d (8.9)	122.9	5.01 d (9.6)	126.8
3		137.1		137.6		137.3
4	2.34 m	38.9	1.58 t (11.7)	39.3	2.92 dd (17.0, 6.9)	36.7
			2.15 dd (11.7, 8.3)		2.46 ddd (17.2, 5.5, 2.1)	
5	2.47 td (12.4, 8.3)	25.1	2.25 td (12.2, 8.3)	23.7	5.87 dddd (11.0, 6.9, 5.5, 1.8)	132.7
	2.31 m		2.01 dt (12.2, 8.3)			
6	6.09 ddq (12.4, 3.4, 1.4)	146.5	5.40 tq (8.3, 1.4)	130.2	5.39 ddd (10.8, 7.6, 2.1)	132.0
7		135.1		140.0	3.13 dq (7.6, 7.3)	47.0
8		206.6		211.5		215.7
9	2.77 t (11.7)	37.1	2.32 d (8.9)	36.3	2.30 dd (15.0, 6.1)	34.6
	2.37 dd (11.7, 2.8)				2.14 dd (15.1, 9.6)	
10	0.77 ddd (11.7, 8.9, 2.8)	29.6	1.38 q (8.9)	28.3	1.39 ddd (9.6, 9.6, 6.1)	30.1
11		20.2		21.7		22.2
12	1.12 s	15.9	1.11 s	16.1	1.10 s	15.2
13	1.08 s	29.3	1.11 s	29.2	1.09 s	28.4
14	1.74 s	13.3	1.88 s	21.9	1.16 d (7.3)	19.1
15	1.49 s	16.7	1.55 s	17.4	1.66 s	18.5

^1^ Recorded in CDCl_3_.

**Table 9:** The ^1^H and ^13^C NMR data of compounds **34** and **35**.

No.	34 [49]		35 [49]	
	*δ*_H_ ^1^	*δ*_C_ ^1^	*δ*_H_ ^1^	*δ*_C_ ^1^
1	0.88 dd (10.3, 8.9)	33.8	0.82 dd (10.3, 8.9)	29.9
2	3.99 d (10.3)	70.3	4.17 d (10.3)	83.5
3		150.6		147.4
4	2.39 m	35.5	2.41 m	36.0
	2.30 m			
5	2.39 m	27.7	2.36 dt (17.2, 7.9)	27.2
	2.30 m		2.27 m	
6	5.74 t (7.8)	131.9	5.52 t (8.1)	130.2
7		138.7		138.2
8		209.2		209.4
9	2.66 dd (14.4, 11.0)	38.6	2.60 m	39.6
	2.48 dd (14.4, 1.4)			
10	1.06 ddd (11.0, 8.9, 1.4)	25.2	0.94 td (8.9, 3.4)	24.7
11		18.4		19.2
12	1.13 s	15.3	1.10 s	15.5
13	1.15 s	28.7	1.10 s	28.5
14	1.83 s	21.0	1.84 s	21.2
15	5.23 s	131.1	5.35 s	116.4
	4.93 s		5.15 s	

^1^ Recorded in CDCl_3_.

**Table 10:** The ^1^H and ^13^C NMR data of compounds **36** and **37**.

No.	36 [49]		37 [49]	
	*δ*_H_ ^1^	*δ*_C_ ^1^	*δ*_H_ ^1^	*δ*_C_ ^1^
1	1.03 dd (11.0, 8.9)	31.3	0.87 dd (11.0, 8.9)	28.2
2	3.70 d (11.0)	73.9	4.08 d (11.0)	86.3
3		152.5		149.5
4	2.66 dd (12.4, 6.9)	35.3	2.72 ddd (12.4, 5.5)	36.9
	2.29 td (12.4, 6.2)		2.33 td (12.4, 5.1, 2.1)	
5	2.50 qd (12.4, 6.2)	30.2	2.48 qd (12.4, 5.5)	30.3
	2.39 m		2.39 m	
6	6.14 ddd (12.4, 6.1, 1.4)	137.3	6.13 ddd (12.4, 5.1, 2.1)	137.1
7		137.0		137.0
8		207.3		207.1
9	2.78 t (12.4)	38.1	2.79 t (13.1)	38.4
	2.51 dd (12.4, 2.1)		2.56 dd (13.1, 2.1)	
10	0.68 ddd (12.2, 8.9, 2.4)	28.1	0.73 ddd (13.1, 8.9, 2.1)	28.5
11		18.6		19.6
12	1.15 s	15.2	1.12 s	15.6
13	1.13 s	29.2	1.10 s	28.9
14	1.66 s	13.4	1.69 s	13.3
15	5.27 s	112.7	5.38 s	115.7
	5.00 s		5.24 s	

^1^ Recorded in CDCl_3_.

**Table 11:** The ^1^H and ^13^C NMR data of compound **39**.

No.	39 [71]			
	*δ*_H_ ^1^	*δ*_C_ ^1^	*δ*_H_ ^2^	*δ*_C_ ^2^
1	5.40 dd (11.0, 5.1)	136.7	5.08 dd (10.3, 4.8)	136.4
2	2.28 dt (14.6, 11.0)	23.7	2.61 m	24.1
	1.98 m		1.94 m	
3	1.75 m	41.9	1.49 dd (14.4, 8.9)	42.1
	1.51 dd (14.6, 11.0)		1.16 dd (14.4, 11.0)	
4		73.9		73.4
5	5.31 d (15.1)	139.5	5.05 d (15.1)	139.7
6	5.33 dd (15.1, 10.3)	124.1	5.55 dd (15.1, 10.3)	124.5
7	3.02 m	47.4	2.94 m	47.8
8	2.84 dd (12.4, 5.5)	44.5	2.69 dd (12.4, 5.5)	44.7
	2.38 t (12.4)		2.51 t (12.4)	
9		207.3		205.0
10		138.1		138.2
11		145.9		146.3
12	1.76 s	20.9	1.69 s	20.9
13	4.79 s	110.3	4.89 s	110.2
	4.78 s		4.85 s	
14	1.95 s	20.0	1.65 s	19.9
15	1.27 s	29.1	1.12 s	29.3

^1^ Recorded in CDCl_3_. ^2^ Recorded in C_6_D_6_.

**Table 12:** The ^1^H and ^13^C NMR data of compounds **42** and **43**.

No.	42 [47]		43 [47]	
	*δ*_H_ ^1^	*δ*_C_ ^1^	*δ*_H_ ^1^	*δ*_C_ ^1^
1	7.88 s	145.42	7.88 s	145.42
2		120.63		120.63
3	6.49 s	106.74	6.49 s	106.74
4		154.61		154.61
5	6.05 br s	113.65	6.05 br s	113.65
6		140.50		140.50
7	2.22 t (7.9)	40.66	2.24 t (7.9)	40.27
8	2.36 q (7.5)	25.80	2.33 q (7.9)	26.70
9	5.37 br t (7.3)	129.75	5.47 br t (7.2)	131.75
10		132.84		132.84
11	6.76 ddd (17.3, 10.8, 0.9)	133.46	6.35 dd (17.1, 10.7)	141.34
12	5.29 br d (17.3)	113.77	5.09 d (17.3)	110.84
	5.09 dt (10.5, 1.5)		4.93 d (10.9)	
13		163.38		163.38
14	1.96 d (1.1)	18.71	1.97 d (1.1)	18.71
15	1.80 q (1.1)	19.74	1.74 s	11.69
16	4.29 q (7.1)	60.38	4.29 q (7.1)	60.38
17	1.34 t (7.2)	14.11	1.34 t (7.2)	14.32

^1^ Recorded in CDCl_3_.

**Table 13:** The ^1^H and ^13^C NMR data of compound **44**.

No.	44 [47]	
	*δ*_H_ ^1^	*δ*_C_ ^1^
1		73.69
2	1.69 dd (13.8, 8.4)	26.75
	1.79 ddd (13.8, 10.4, 8.3)	
3	1.64 ddddq (12.2, 10.4, 8.4, 7.6, 0.5)	26.72
	1.12 dd (12.0, 8.3)	
4	2.37 dq (7.6, 7.4)	37.60
5		69.34
6	3.05 s	58.08
7		68.59
8	1.87 dddd (15.6, 6.3, 4.0, 0.9)	22.44
	1.91 ddd (15.6, 10.8, 4.1)	
9	1.77 dddd (14.6, 10.8, 4.2, 4.0)	26.27
	1.23 dddd (14.6, 7.5, 6.3, 4.1)	
10	2.36 dqdd (7.5, 7.2, 4.2, 0.9)	31.45
11	1.49 qq (7.0, 6.8)	36.50
12	0.92 d (7.0)	17.81
13	0.969 d (6.8)	17.86
14	1.02 d (7.2)	17.30
15	0.974 dd (7.4, 0.5)	16.01

^1^ Recorded in CDCl_3_.

**Table 14:** The ^1^H and ^13^C NMR data of compounds **45** and **46**.

No.	45 [45]		46 [45]	
	*δ*_H_ ^1^	*δ*_C_ ^1^	*δ*_H_ ^1^	*δ*_C_ ^1^
1	5.75 d (3.6)	92.3	5.87 d (3.3)	92.0
2	/	/	/	/
3	6.52 d (1.9)	142.6	6.51 d (1.3)	142.4
4		116.1		116.2
4a	2.29 m	39.7	2.38 m	37.0
5	2.08 m	32.2	2.41 m	31.2
	1.22 m		2.23 m	
6	2.19 m	39.7	2.19 m	39.2
	2.07 m		1.19 m	
7		134.4		59.8
8	5.73 dd (9.5, 8.0)	124.3	2.98 dd (9.5, 8.0)	62.3
9	2.43 m	25.5	2.22 m	25.3
	2.07 m		1.43 m	
10	2.31 m	35.9	1.35 m	31.2
	2.07 m			
11		149.3		146.8
11a	1.96 br s	50.1	2.43 m	49.3
12	5.25 t (7.6)	74.7	5.24 t (7.6)	74.3
13	2.43 m	31.4	2.41 m	31.3
	2.31 m		2.23 m	
14	4.97 t (6.9)	118.9	4.95 m	118.6
15		135.6		134.7
16	1.64 br s	18.1	1.61 s	18.1
17	1.67 br s	25.7	1.67 s	25.7
18	1.67 br s	17.0	1.31 s	17.2
19	4,91 br s	113.4	5.06 br s	116.2
	4.78 br s		4.93 br s	
CO*CH_3_*	2.07 s	21.5	2.08 s	21.4
*CO*CH_3_		169.5		170.2
CO*CH_3_*	2.01 s	21.0	2.00 s	21.0
*CO*CH_3_		170.2		169.3

^1^ Recorded in CDCl_3_.

**Table 15:** The ^1^H and ^13^C NMR data of compounds **47** and **48**.

No.	47 [45]		48 [45]	
	*δ*_H_ ^1^	*δ*_C_ ^1^	*δ*_H_ ^1^	*δ*_C_ ^1^
1	6.14 d (2.3)	93.4	6.12 d (2.3)	93.3
2	/	/	/	/
3	7.25 d (2.0)	151.7	7.26 d (2.0)	151.2
4		124.3		123.5
4a	3.44 m	28.2	3.32 m	28.6
5	1.73 m	27.0	1.69 m	26.9
	1.57 m		1.55 m	
6	2.89 m	37.5	2.84 br t (12.2)	37.8
	1.46 m		1.43 m	
7		83.7		83.5
8	5.40 d (11.9)	131.2	5.41 d (11.8)	131.4
9	5.59 m	129.8	5.57 m	129.7
10	3.17 m	30.7	3.16 m	30.8
	2.63 m		2.65 m	
11		145.5		145.6
11a	2.63 m	48.1	2.60 d (12.3)	48.0
12		190.2		197.7
13	6.51 d (15.8)	125.6	3.25 t (5.6)	38.6
14	6.82 d (15.8)	148.4	5.30 t (7.0)	117.0
15		82.1		135.0
16	1.40 s	24.2	1.74 d (1.0)	25.8
17	1.39 s	24.1	1.64 s	18.1
18	1.61 s	28.5	1.59 s	28.5
19	5.14 d (7.0)	118.1	5.12 d (8.3)	117.9
CO*CH_3_*	2.06 s	22.3	2.04 s	22.2
*CO*CH_3_		169.2		169.3
CO*CH_3_*	2.04 s	20.9	2.04 s	20.9
*CO*CH_3_		169.2		169.1

^1^ Recorded in CDCl_3_.

**Table 16:** The ^1^H and ^13^C NMR data of compounds **50**, **51,** and **53**.

No.	50 [47]		51 [47]		53 [47]	
	*δ*_H_ ^1^	*δ*_C_ ^1^	*δ*_H_ ^1^	*δ*_C_ ^1^	*δ*_H_ ^1^	*δ*_C_ ^1^
1	1.80–1.90 m	37.24		37.25		37.23
	1.03–1.10 m					
2	1.80–1.90 m	31.65		31.67		31.61
	1.47–1.60 m					
3	3.52 tt (11.2, 4.6)	71.82	3.52 tt (11.2, 4.6)	71.81	3.52 tt (11.2, 4.6)	71.92
4	2.29 ddd (12.8, 4.9, 1.9)	42.28		42.31		42.23
	2.19–2.26 m					
5		140.73		140.76		140.68
6	5.34 dt (5.0, 2.0)	121.71	5.36 dt (5.3, 2.0)	121.73	5.35 dt (5.0, 2.2)	121.80
7	1.94–2.03 m	31.89		31.90		31.86
	1.47–1.60 m					
8	1.40–1.47 m	31.89		31.90		31.96
9	0.90–0.94 m	50.11		50.12		50.14
10		36.49		36.50		36.51
11	1.47–1.60 m	21.07		21.08		21.07
	1.40–1.47 m					
12	1.94–2.03 m	39.76		39.76		39.85
	1.10–1.19 m					
13		42.35		42.31		42.76
14	0.99–1.01 m	56.75		56.75	1.00 br s	56.49
15	1.47–1.60 m	24.28		24.29		24.51
	1.03–1.10 m					
16	1.80–1.90 m	28.21		28.19		28.21
	1.25–1.30 m					
17	1.10–1.19 m	55.97		55.98		57.90
18	0.67 s	11.85	0.66 s	11.85	0.65 s	11.90
19	1.00 s	19.39	1.00 s	19.40	1.00 s	19.40
20	1.40–1.47 m	35.74		36.18	0.98–1.01 m	35.28
21	0.94 d (6.4)	18.70	0.91 d (6.8)	18.88	0.98–1.01 m	21.17
22	1.47–1.60 m	34.67		33.71	0.13–0.19 m	32.03
	1.10–1.19 m					
23_3_	2.05–2.11 m	30.96		30.56		25.80
	1.80–1.90 m					
24		156.89		39.06	0.24 dqd (8.8, 7.0, 1.8)	50.80
25	2.19–2.26 m	33.79		31.45		32.14
26	1.011 d (6.8)	21.86	0.77 d (6.8)	17.58	0.85 d (6.4)	21.53
27	1.014 d (6.8)	21.99	0.84 d (6.8)	20.52	0.93 d (7.5)	22.18
28	4.64 br d (1.5)	105.91	0.76 d (6.8)	15.44	0.94 d (6.9)	15.45
	4.70 br s					
29					0.45 ddd (9.1, 4.3, 2.6)	21.29
					–0.14 ddd (5.8, 4.4, 1.3)	
30					0.89 s	14.27

^1^ Recorded in CDCl_3_.

**Table 17:** The ^1^H and ^13^C NMR data of compounds **56**–**58**.

No.	56 [45]		57 [45]		58 [45]	
	*δ*_H_ ^1^	*δ*_C_ ^1^	*δ*_H_ ^1^	*δ*_C_ ^1^	*δ*_H_ ^1^	*δ*_C_ ^1^
1	7.13 d (10.2)	158.5	7.12 d (10.2)	158.0	6.18 d (10.0)	142.0
2	5.83 d (10.0)	127.4	5.85 d (10.2)	127.5	5.60 ddd (10.0, 5.5, 1.2)	122.5
3		200.1		200.0	5.15 m	67.3
4	2.35 dd (17.7, 14.1)	40.1	2.36 dd (17.6, 14.1)	40.1	1.81 m	31.8
	2.20 dd (17.7, 3.6)		2.21 dd (17.7, 4.0)		1.53 m	
5	1.93 m	44.4	1.95 m	44.4	1.58 m	39.6
6	1.78 m	27.2	1.45 m	27.5	1.37 m	27.6
	1.55 m		1.41 m		1.37 m	
7	1.74 m	31.4	1.67 m	31.2	1.68 m	31.7
	0.99 m		0.98 m		1.02 m	
8	1.45 m	35.8	1.47 m	35.3	1.40 m	35.3
9	0.97 m	50.3	1.02 m	50.3	0.99 m	51.0
10		39.1		39.1		37.9
11	1.42 m	27.6	1.85 m	33.8	1.85 m	33.9
			1.56 m		1.58 m	
12	1.76 m	37.4	1.74 m	37.2	1.71 m	37.3
	1.07 m		1.20 m		1.14 m	
13		43.7		44.2		44.2
14	1.08 m	55.6	1.38 m	53.3	1.37 m	53.4
15	1.76 m	20.8	1.77 m	20.4	1.76 m	20.2
	1.42 m		1.42 m		1.37 m	
16	1.66 m	24.7	5.11 m	78.4	5.08 m	78.5
	1.18 m					
17	1.95 m	55.3	2.15 dd (7.7, 4.1)	61.6	2.13 t (7.7)	61.6
18	0.61 s	13.0	0.69 s	14.2	0.66 s	14.2
19	1.00 s	13.0	1.01 s	13.1	0.81 s	13.9
20	5.74 td (16.5, 10.8, 8.8)	139.5	5.77 td (17.1, 10.5, 8.9)	135.9	5.76 td (17.1, 10.4, 8.9)	136.1
21	4.95 m	114.7	5.08 m	117.0	5.04 m	116.8
CO*CH_3_*			2.02 s	21.2	2.04 s	21.3
*CO*CH_3_				171.1		171.1
CO*CH_3_*					2.02 s	21.5
*CO*CH_3_						170.1

^1^ Recorded in CDCl_3_.

**Table 18:** The ^1^H and ^13^C NMR data of compounds **59**–**61**.

No.	59 [50]		60 [50]		61 [50]	
	*δ*_H_ ^1^	*δ*_C_ ^1^	*δ*_H_ ^1^	*δ*_C_ ^1^	*δ*_H_ ^1^	*δ*_C_ ^1^
1	1.79 m	36.9	1.93 m	33.5	1.41 m	31.4
	1.12 m					
2	1.82 m	31.2	1.83 m	31.5	1.81 m	31.5
	1.48 m		1.35 m		1.95 m	
3	3.55 m	71.2	3.63 m	71.1	3.49 m	71.6
4	2.32 ddd (12.2, 4.9, 1.5)	41.8	2.44 ddd (11.4, 5.6, 1.5)	41.9	2.28 ddd (11.2, 5.6, 1.5)	42.1
	2.23 ddd (12.2, 11.3)		2.26 brdd (11.6, 11.4)		2.20 ddd (11.6, 11.2)	
5		146.0		141.3		140.6
6	5.59 dd (5.3, 1.5)	123.9	5.95 dd (5.4, 1.5)	128.6	5.34 br s	121.5
7	3.85 br s	64.9	3.91 dd (3.5, 1.9)	64.7	1.92 m	26.2
					1.54 m	
8	1.39 m	36.5	1.89 m	37.9	1.25 m	31.0
9	1.41 m	41.5	1.44 m	41.6	1.08 m	54.9
10		37.5		42.2		36.6
11	1.87 m	27.0	1.90 m	27.5	1.77 m	37.2
	1.40 m		1.66 m		1.07 m	
12	4.63 dd (9.8, 4.5)	80.8	4.61 dd (11.1, 4.6)	80.8	4.66 dd (10.7, 4.6)	81.1
13		46.1		46.4		46.2
14	1.56 m	57.4	1.55 m	57.4	1.10 m	49.0
15	1.79 m	23.6	1.76 m	23.4	1.65 m	23.7
	1.24 m		1.24 m		1.20 m	
16	1.94 m	27.9	1.56 m	27.9	1.80 m	27.6
	1.56 m				1.40 m	
17	1.53 m	48.3	1.43 m	49.4	1.52 m	55.1
18	0.80 s	9.0	0.86 s	9.2	0.80 s	8.5
19	0.97 s	18.1	3.90 d (11.1)	63.1	1.00 s	19.3
			3.60 d (11.1)			
20	1.15 m	33.6	1.16 m	33.6	1.52 m	39.0
21	0.93 d (6.8)	22.2	0.95 d (6.8)	22.2	0.86 d (6.6)	13.6
22	0.24 ddd (8.7, 5.4, 5.1)	30.6	0.23 ddd (8.7, 5.4, 5.2)	30.6	1.53 m	41.1
					0.98 m	
23		25.3		25.3	3.81 dd (9.2, 2.5)	70.8
24	0.23 m	50.6	0.23 m	50.7	1.54 m	32.7
25	1.55 m	32.2	1.55 m	32.2	1.48 m	35.0
26	0.84 d (6.8)	21.5	0.84 d (6.8)	21.5	0.82 d (6.6)	15.2
27	0.93 d (6.8)	22.2	0.93 d (6.8)	22.2	0.85 d (6.6)	20.1
28	0.92 d (6.8)	15.4	0.91 d (7.2)	15.4	0.80 d (6.6)	18.0
29	0.45 dd (8.7, 4.5)	21.5	0.45 dd (8.7, 4.3)	21.5		
	–0.13 dd (5.1, 4.5)		–0.13 dd (5.4, 4.5)			
30	0.89 s	13.8	0.90 s	13.8		
CO*CH_3_*	2.01 s	21.8	2.02 s	21.8	2.01 s	21.7
*CO*CH_3_		170.7		170.8		170.5

^1^ Recorded in CDCl_3_.

**Table 19:** The ^1^H and ^13^C NMR data of compounds **62** and **63**.

No.	62 [50]		63 [50]	
	*δ*_H_ ^1^	*δ*_C_ ^1^	*δ*_H_ ^1^	*δ*_C_ ^1^
1	1.93 m	26.3	1.63 m	26.8
2	1.84 m	31.4	1.83 m	31.3
3	3.51 m	71.3	3.52 m	71.1
4	2.28 m	41.3	2.29 m	41.3
5		141.5		140.6
6	5.23 br s	120.4	5.27 br s	121.0
7	2.16 m	31.7	2.17 m	31.4
	1.55 m		1.46 m	
8	1.87 m	27.2	1.86 m	27.4
9	1.12 m	53.2	1.20 m	52.2
10		36.9		36.2
11	4.26 dd (3.2, 3.2)	70.4	5.53 dd (3.3, 3.3)	73.4
12	4.64 dd (3.2)	82.7	3.52 d (3.3)	80.0
13		45.9		47.2
14	1.10 m	57.2	1.01 m	57.3
15	1.55 m	41.1	1.55 m	41.0
	1.01 m		1.02 m	
16	3.81 m	70.7	3.80 ddd (9.7, 3.6, 1.3)	72.4
17	1.54 m	55.1	1.50 m	56.7
18	1.01 s	10.7	0.86 s	10.2
19	1.26 s	22.6	0.97 s	21.9
20	1.45 m	39.6	1.59 m	39.7
21	0.87 d (6.8)	13.5	0.98 d	12.1
22	1.72 m	23.6	1.71 m	23.3
23	1.93 m	36.5	1.84 m	37.1
	1.19 m		1.15 m	
24	1.48 m	35.0	1.48 m	35.0
25	1.55 m	32.7	1.55 m	32.7
26	0.81 d (6.8)	18.0	0.81 d (6.6)	17.9
27	0.86 d (6.8)	20.1	0.85 d (6.6)	20.1
28	0.82 d (6.8)	15.2	0.81 d (6.6)	15.1
CO*CH_3_*	2.12 s	21.7	2.10 s	21.8
*CO*CH_3_		170.0		174.5

^1^ Recorded in CDCl_3_.

**Table 20:** The ^1^H and ^13^C NMR data of compounds **64**–**66**.

No.	64 [46]		65 [46]		66 [76]	
	*δ*_H_ ^1^	*δ*_C_ ^1^	*δ*_H_ ^1^	*δ*_C_ ^1^	*δ*_H_ ^1^	*δ*_C_ ^1^
1	7.04 d (10.2)	157.0	7.06 d (10.2)	157.0	1.73 m	37.1
					2.04 m	
2	6.21 dd (10.2, 1.8)	126.7	6.22 d (10.2, 1.8)	126.7	1.58 m	32.0
3		186.6		186.6	1.26 m	38.5
					2.03 m	
4	6.16 d (1.8)	125.9	6.17 d (1.8)	186.6	4.36 s	73.3
5		165.7		125.9		168.3
6	4.55 br s	74.0	4.55 br s	165.7	5.82 s	126.3
7	1.30 m	40.0	2.09 m	74.0		202.0
	2.07 m		2.28 m			
8	2.05 m	30.3	2.07 m	30.4	2.53 m	39.6
9	1.12 m	51.3	1.13 m	51.3	0.91 m	53.6
10		43.5		43.5		38.0
11	1.74 m	22.6	1.75 m	22.6	1.50 m	22.2
12	1.72 m	31.6	1.76 m	31.6	1.17 m	39.6
					2.05 m	
13		47.7		47.7		42.6
14	1.69 m	49.6	1.79 m	49.6	1.35 m	50.8
15	1.24 m	23.6	1.25 m	23.6	1.65 m	24.1
16	1.85 m	38.1	1.88 m	38.1	1.89 m	28.1
17		85.4		85.4	1.16 m	55.9
18	0.81 s	14.6	0.84 s	14.6	0.75 s	12.0
19	1.44 s	20.4	1.45 s	20.4	1.38 s	19.5
20	1.95 t (7.2)	41.2	1.74 m	41.2	1.43 m	35.8
21	1.02 d (7.2)	8.7	1.01 d (7.2)	8.7	0.96 d (6.6)	18.7
22	5.32 t (7.2)	74.0	4.18 br s	74.0	1.16 m	31.0
					1.53 m	
23	2.18 m	39.2	2.12 m	39.2	1.86 m	34.6
	2.40 dd (13.8, 7.2)		2.28 m		2.18 m	
24		151.5		151.5		156.8
25	2.25 t (6.6)	33.4	2.25 t (7.2)	33.4	2.21 m	33.8
26	1.04 d (6.6)	21.7	1.07 d (7.2)	21.7	1.02 d (3.0)	21.9
27	1.05 d (6.6)	21.8	1.05 d (7.2)	21.8	1.03 d (3.0)	22.0
28	4.75 s	110.2	4.80 s	110.2	4.66 s	106.0
	4.86 s		4.93 s		4.72 s	
CO*CH_3_*	2.02 s	21.4				
*CO*CH_3_		171.4				

^1^ Recorded in CDCl_3_.

## Data Availability

Not applicable.
